# Elucidating the anticancer activities of guanidinium-functionalized amphiphilic random copolymers by varying the structure and composition in the hydrophobic monomer

**DOI:** 10.7150/thno.60711

**Published:** 2021-08-21

**Authors:** Joyce Tay, Yanli Zhao, James L. Hedrick, Yi Yan Yang

**Affiliations:** 1 Institute of Bioengineering and Bioimaging, 31 Biopolis Way, The Nanos, Singapore 138669, Singapore.; 2 Division of Chemistry and Biological Chemistry, School of Physical and Mathematical Sciences, Nanyang Technological University, 21 Nanyang Link, Singapore 637371, Singapore.; 3 IBM Almaden Research Center, 650 Harry Road, San Jose, California 95120, United States.

**Keywords:** Anticancer polymers, Guanidinium-functionalized polycarbonates, Hydrophobicity, Biodegradability, Functional mechanism

## Abstract

**Rationale:** Use of traditional anticancer chemotherapeutics has been hindered by the multifactorial nature of multi-drug resistance (MDR) development and metastasis. Recently, cationic polycarbonates were reported as novel unconventional anticancer agents that mitigated MDR and inhibited metastasis. The aim of this study is to explore structure-anticancer activity relationship. Specifically, a series of cationic guanidinium-based random copolymers of varying hydrophobicity was synthesized with a narrow polydispersity (Ð = 1.12-1.27) *via* organocatalytic ring-opening polymerization (OROP) of functional cyclic carbonate monomers, and evaluated for anticancer activity, killing kinetics, degradability and functional mechanism.

**Methods:** Linear, branched and aromatic hydrophobic side chain units, such as ethyl, benzyl, butyl, isobutyl and hexyl moieties were explored as comonomer units for modulating anticancer activity. As hydrophobicity/hydrophilicity balance of the polymers determines their anticancer efficacy, the feed ratio between the two monomers was varied to tune their hydrophobicity.

**Results:** Notably, incorporating the hexyl moiety greatly enhanced anticancer efficiency and killing kinetics on cancer cells. Degradation studies showed that the polymers degraded completely within 4-6 days. Flow cytometry and lactate dehydrogenase (LDH) release analyses demonstrated that anticancer mechanism of the copolymers containing a hydrophobic co-monomer was concentration dependent, apoptosis at IC_50_, and both apoptosis and necrosis at 2 × IC_50_. In contrast, the homopolymer without a hydrophobic comonomer killed cancer cells predominantly *via* apoptotic mechanism.

**Conclusion:** The hydrophobicity of the polymers played an important role in anticancer efficacy, killing kinetics and anticancer mechanism. This study provides valuable insights into designing novel anticancer agents utilizing polymers.

## Introduction

The genesis of chemotherapy was conceived in the 1940s during World War II when the nitrogen mustard gas was unintentionally recognized for its anti-leukemia potential 1. Since then, a plethora of chemotherapeutic drugs have been developed. This revolutionary idea kindled the hope for a prospective elixir for cancer. However, a promise is almost all it held, as the magical efficacy bestowed upon the cancer patients was only initial and remission occurred in most of them. Research focused on discovering effective anticancer agents has yet to succeed in achieving its goal. Only in the last decades has imatinib (Glivec) been developed as a promising agent against chronic myelogenous leukemia 2. Antitumor drugs that are effective against an extensive spectrum of tumors are not available, and ideal drugs that act against these tumors have yet to be discovered.

Poor efficacy and significant toxicity of the anticancer drugs presented in the market are some of the major challenges haunting the present treatment modalities available. One such notorious example involved the molecular target drug, rofecoxib, with over US$10 billion being compensated due to the onset of unforeseen detrimental effects 3. Supposedly, conventional chemotherapy agents are expected to be highly selective towards cancer cells that proliferate rapidly. Unfortunately, they cause non-specific toxicity to normal cells 4, 5. Above all, the intrinsic adversity in cancer therapy remains in the great genetic variations and mutations characteristics inherent of human cancers 6, thereby leading to multidrug resistance (MDR) as treatment progressed along the way 7-10.

Having identified and recognized the constraint posted by small molecule drugs, the burgeoning focus on peptide therapeutics by the scientific community is not solely a matter of pure coincidence. Indeed, after much judicious investigation, it was discovered that host defense peptides (HDPs) and synthetic antimicrobial peptides (AMPs), which were synthesized with the original intention to kill bacteria, are equally promising against cancer cells 11. The inherited characteristics of these amphiphilic, cationic peptides facilitate their interaction with the negatively charged bacterial membrane. Analogously, the presence of phosphatidylserine, sialic acid, or heparin sulphate also contributed to the difference in the negative charge density of the cancer cell membrane as compared to their healthy counterparts 12, which facilitated the accumulation of the cationic AMPs selectively on the membrane surface of the cancer cells electrostatically and mediated membrane disruption via hydrophobic interaction with the lipophilic cell membrane 13, 14. Despite the high cytotoxicity of these oncolytic peptides towards cancer cells, coupled with their ability to target multiple sites via numerous mechanisms to escape the development of MDR, prevailing challenges such as soaring manufacturing costs deprive them of clinical applications 15.

Achieving “amphiphilic balance”, which is the optimization of the balance between cationic charge and hydrophobicity, is of uttermost paramount for the rational design of AMPs 16-20. Nonetheless, despite their ability to bind to anionic cell surface electrostatically, the challenge remains for these highly cationic peptides to insert themselves into the hydrophobic region of the membrane. On the other end of the spectrum, the use of overly hydrophobic peptides led to the occurrence of unselective binding and insertion into human cell membrane even in the absence of electrostatic attraction. Such actions are indiscriminately toxic to both healthy and cancerous cells 21, 22. Only when the cationic and hydrophobic moieties are present in the appropriate ratio, the peptides can selectively bind to the targets of interest and insert preferable into the target cell membrane as compared to healthy cells. Various strategies have been reported to tune the amphiphilicity of polymers, such as introducing the hydrophobic moiety as separate monomers (segregated polymer approach) or attaching it directly to the cationic moiety (same-centered polymer approach) 23. The former strategy provides greater flexibility to vary the identity and number of repeating units in order to fine tune the hydrophobic/hydrophilic balance of the polymers, independently of the cationic moiety.

Since the last decades, many research groups have delved into the potential of synthetic amphiphilic cationic polymers as antimicrobial agents 23. Nevertheless, the non-biodegradable backbone in these polymers might cause toxicity to the body due to long-term accumulation. Polycarbonates are considered to be an attractive class of biomaterials due to their excellent biocompatibility and biodegradability 24-28. Recently, we developed and applied a living organocatalytic ring-opening polymerization (OROP) approach to synthesize biodegradable polycarbonate-based antimicrobial macromolecules which resulted in precisely controlled molecular lengths and narrow molecular weight distribution 29-34. Such approach remarkably favors the study of structure-activity relationship. These guanidinium-functionalized polycarbonates could translocate across the bacterial cell membrane whilst ensuring that the membrane remained intact, precipitating cytosolic biomolecules 35.

**Scheme 1:**
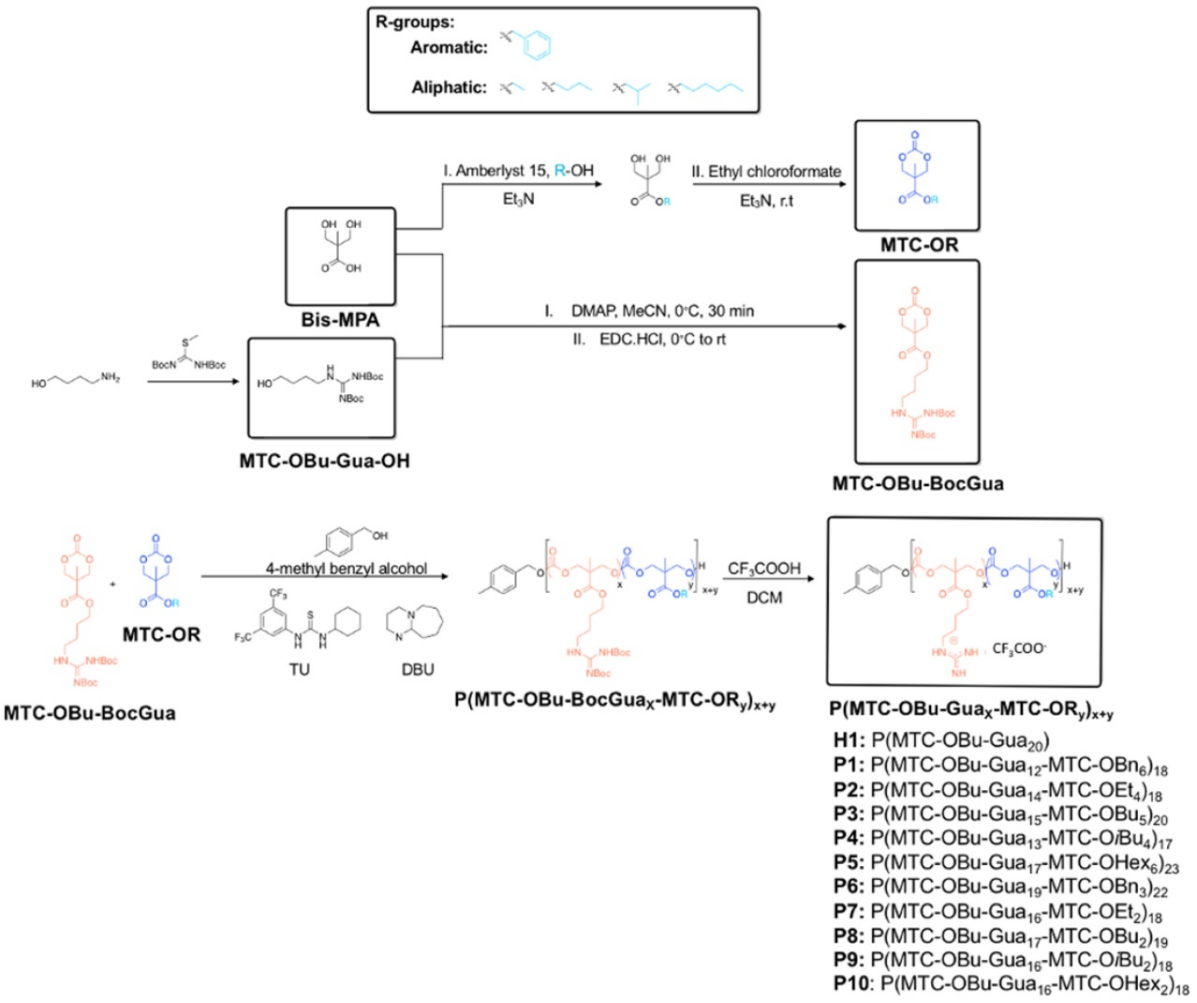
General synthetic procedures and chemical structure of polycarbonate-based random amphiphilic copolymers with cationic guanidinium and hydrophobic side chains. The composition of hydrophobic monomer was varied, with R ranging from the aromatic benzyl, to aliphatic ethyl, butyl, isobutyl and hexyl groups.

**Figure 1:**
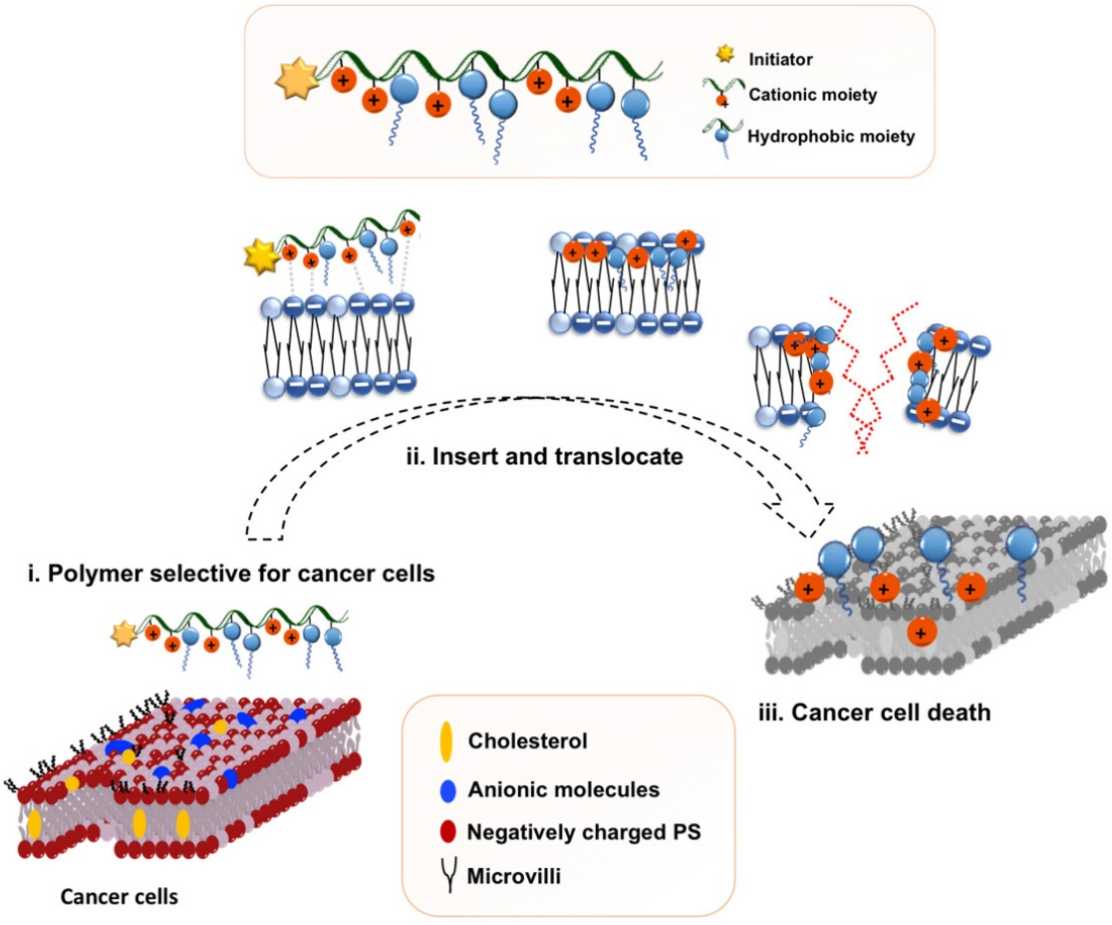
Proposed polymeric design and initial hypothesis on mechanism of action of the amphiphilic copolymer.

Recently, we reported *in vitro* and *in vivo* anticancer activity of biodegradable cationic polycarbonate nanoparticles formed from quaternary ammonium-containing polycarbonates 36 and guanidinium-functionalized polycarbonates 37. Bidentate hydrogen bonded ion pairing between the guanidinium group present in the polymer and the phosphate lipid head groups on the cell membrane facilitated the formation of a complex. It is hypothesized that such affinity for phospholipids is accountable for promoting polymer-membrane interaction and the resulting cell-penetrating ability, as well as imparting selectivity to cancer cells over healthy cells 37. Analogous to their quaternary ammonium counterparts, the introduction of other functionalities onto the guanidinium polymers, whilst ensuring the charge density remains above 50 mol%, did not jeopardize the polymers' antimicrobial activity 38. Furthermore, the hydrophilic/hydrophobic balance of these co-polymers can be tuned by varying the spacer length between the guanidine group and the polycarbonate backbone 39. However, no exhaustive study has been performed on tuning their hydrophilic/hydrophobic balance by varying their hydrophobic moiety and attesting their anticancer activity. Hence, in this study, we intended to explore structure-anticancer activity relationship and anticancer mechanism. Specifically, a series of guanidinium-functionalized amphiphilic random copolymers was synthesized with various hydrophobic groups (Scheme 1). The ethyl, butyl, isobutyl, hexyl and benzyl side chains of the hydrophobic monomers serve as the hydrophobic groups to balance polymer amphiphilicity (Figure 1). Particularly, two main strategies were employed in the design of the polymers in an attempt to tune amphiphilicity: adjusting the (1) ratio of hydrophobic and cationic groups and (2) the identity of the hydrophobic moieties in the copolymers. The resulting anticancer activity was evaluated against HepG2 human liver, MCF-7 human breast and SW480 human colorectal cancer cell lines and analyzed using alarmaBlue assay. Morphological changes to the cells served as indicators of cell survival by microscopic investigations; the release of the cytosolic enzyme lactate dehydrogenase (LDH) was used to quantify the extend of membrane damage since cationic macromolecules potentially interact with the cell membrane and can provide knowledge on the effects of polymers on polymer-membrane interaction. Finally, we systematically investigated the nature of cytotoxic reaction caused by these random copolymers with respect to apoptotic or necrotic mechanisms using confocal microscopy and flow cytometry.

## Materials and methods

### Materials

All anhydrous solvents were purchased from Sigma-Aldrich or Merck. All solvents used were of HPLC or analytical grade and were purchased from Fisher Scientific, J.T. Baker, VWR or Fulltime. No further purification was done upon received. 1,8-Diazabicyclo[5.4.0]undec-7-ene (DBU; 98%) was stirred over CaH_2_ and vacuum distilled before storing in a glove box. Cyclic carbonate monomer bearing an ethyl, butyl, isobutyl, hexyl and benzyl group (MTC-OR, R: hydrophobic group), Boc-protected butyl guanidine group (MTC-OBu-BocGua), N-(3,5-trifluoromethyl)phenyl-N'-cyclohexylthiourea (TU), and guanidinium-functionalized polycarbonate homopolymer (degree of polymerization-DP: 20) were synthesized according to our previous protocols 29, 35, 40. *N,N'*-Carbonyldiimidazole (CDI) was purchased from Oakwood Chemical and used as received. They were freeze-dried under high vacuum before being transferred to the glove box. All polymers were lyophilized prior to usage in biological studies. HepG2, MCF-7 and SW480 cell lines were obtained from ATCC (U.S.A). The cells were maintained in Dulbecco's modified Eagle medium (DMEM) bought from Invitrogen (Singapore) and Thermo Fisher Scientific, respectively. Phosphate-buffered saline (PBS) was purchased from 1^st^ BASE (Singapore). AlamarBlue Cell Viability Reagents and Image-IT^TM^ Live Plasma Membrane and Nuclear Labelling Kit were purchased from ThermoFisher Scientific, Singapore. CytoTox 96 Non-Radioactive Cytotoxicity Assay was purchased from Promega Corporation and used according to the manufacturer's protocol.

### Polymer characterization

The polymers were characterized for ^1^H NMR spectra using a Bruker Advance 400 spectrometer (400 MHz) at 22 ºC in DMSO, CDCl_3_ or CD_3_OD. Chemical shift values (δ) are reported in ppm, with the solvent signal serving as the internal reference. Molecular weight distribution of the Boc-protected polymers was measured *via* size exclusive chromatography (SEC) according to a previously reported protocol 35.

Critical micelle concentration (CMC) of the polymers in PBS was examined using the fluorescence spectroscopy as per a previously reported protocol 41.

### Synthesis of cyclic carbonate monomers

The cyclic carbonate monomers were synthesized according to a previously published protocol and the brief description of the experimental procedures can be found in the supplementary information 29, 40, 42.

### Synthesis of Boc-protected guanidinium-functionalized random copolymers P(MTC-OBu-BocGua_x_-co-MTC-OR_y_)_x+y_

The Boc-protected guanidinium-functionalized random co-polymers were synthesized according to the previously published protocol 35. The synthesis of **P5'** is given as a representative example. In a nitrogen-filled glovebox, a mixture of 4-methylbenzyl alcohol (5.09 mg, 0.040 mmol), MTC-OBu-BocGua (355.1 mg, 0.75 mmol), MTC-OHex (61.1 mg, 0.25 mmol), thiourea catalyst (3.72 mg, 0.010 mmol), and DBU (1.52 μL, 0.010 mmol) was reacted for 15 min. Once completed, the mixture was quenched with a minute amount of benzoic acid. Purification of the crude product was done via a Sephadex LH-20 column using THF as the eluent. Puffy white solid was obtained as the product upon complete removal of the solvent in vacuo. **P5':** 86% yield, ^1^H NMR (400 MHz, CHCl_3_, 22 ºC): δ 11.50 (s, 12H, N*H* ), 8.38 (s, 15H, N*H*), 4.29 (m, 80H, -C*H*_a_H_b_ and -CH_a_*H*_b_ and -OC*H*_2_ of hydrophobic moiety), 4.16-4.09 (m, 43H, -OC*H*_2_), 3.47-3.42 (m, 33H, -C*H*_2_N), 2.34 (s, 3H, initiator -C*H*_3_), 1.69-1.59 (m, 98H, -C*H*_2_C*H*_2_- and -OCH_2_C*H*_2_ of hydrophobic moiety ), 1.49 (s, 327H, Boc -C*H*_3_), 1.34-1.19 (m, 95H, -C*H*_2_C*H*_2_C*H*_2_- of hydrophobic moiety), 0.88 (t, J = 6.64 Hz, 17H, terminal alkyl -C*H*_3_).

### Synthesis of deprotected guanidinium-functionalized random copolymers P(MTC-OBu-Gua_x_-co-MTC-OR_y_)_x+y_

The post-polymerization removal of Boc groups was done according to previously published protocol 35. Briefly, DCM (9 mL) was used to dissolve P(MTC-OBu-BocGua_x_-*co*-MTC-OR_y_)_x+y_ and trifluoroacetic acid (1 mL) was added and stirred at room temperature overnight. Subsequently, the solvent was removed under high vacuum to yield pale yellow sticky solid as the deprotected guanidinium-functionalized polymer in quantitative yield. The polymer was then dissolved in methanol and precipitated from cold diethyl ether (Et_2_O). The resulting solid was collected following centrifugation and decanting the supernatant thrice to isolate the desired product. After that, the polymer was solubilized in DI water and lyophilized to obtain a white solid. Complete deprotection was confirmed by ^1^H-NMR analysis. **P5** is given as a representative example. **P5:** Yield: 42%, ^1^H-NMR (400 MHz, MeOD, 22 ºC); δ 4.32 (m, 88H, C*H*_a_H_b_ and CH_a_*H*_b_ overlapped with residual H_2_O peak), 4.18 (m, 47 H, -OC*H*_2_-), 3.24 (m, 34H, -C*H*_2_N-), 1.77-1.64 (m, 84H, -C*H*_2_-), 1.41-1.35 (m, 31H, -C*H*_2_ of hexyl side chain), 1.22 (bs, 65H, -C*H*_3_-), 0.93 (t, J = 6.64 Hz, 17H, terminal -C*H*_3_ of hexyl side chain).

### Octanol-water bilayer partitioning of the polymers

Modified dansyl alcohol initiator was used in the synthesis of a series of dansylated guanidinium copolymers (Figure S1). Samples preparation was done according to a previously reported protocol with slight modifications 43. Briefly, the respective polymers were dissolved in PBS to obtain a final stock concentration of 500 µg/mL. An aqueous solution of the polymer in PBS (0.5 mL) were added into a 2 mL Eppendorf tube and topped up with octanol (0.5 mL) to make up to a final volume of 1 mL. The bilayer mixture was vortexed for 5 min and set aside in the absence of light overnight. Subsequently, the bilayer mixture was centrifuged (3000 rpm, 5 min) and each phase was aliquoted out. Dilution (10-fold) was done in methanol before measuring their fluorescence spectra. The concentration of polymer in each phase was then determined with reference to calibration curve, which was plotted by measuring the fluorescence spectra of the respective polymers of varying concentrations in methanol. The partition coefficient was calculated based on log P = log ([P]_oct/_[P]_aq_) where [P]_oct_ and [P]_aq_ are the concentration of the polymer in the octanol and aqueous phases, respectively. All measurements were done in triplicates using Horiba Jobin Yvon Fluoromax 4 fluorospectrometer.

### Study of polymer degradation

To examine the degradation behavior of the polymers, the respective polymer was dissolved in PBS to achieve a final concentration of 10 mg/mL. The solution was store in a 37 ºC oven to simulate the physiological environment. Aliquot amount of the solution (0.5 mL) was taken out periodically at 0 h, 8 h, 24 h, 48 h, 96 h and 144 h, and freeze-dried for ^1^H-NMR analysis. Owning to the gradual disintegration of the carbonate backbone of the polymer, the signal of the methyl protons of the polycarbonate was downshifted. Quantitative analysis of the polymer degradation was then performed based upon the integral intensities of the methyl -C*H*_3_ peaks at 1.30 and 1.18, respectively.

### *In vitro* cytotoxicity study against cancer and healthy cells

AlamarBlue cell viability assay was used to determine the cytotoxicity of polymers against HepG2, MCF-7 and SW480 cancer cell lines as well as HK2 healthy kidney cell line. HepG2, MCF-7, SW480 and HK2 cells were seeded onto a 96-well black plate (density of 5000 cells per well) and left to culture in the incubator under standard conditions for 24 h. The respective polymer was dissolved in fresh RPMI/DMEM to make up various concentrations and pumped into the cells and incubated overnight at 37 ºC. The supernatant was then removed completely before the mixture of alamaBlue and DMEM solution was added to the cells. After 2 h incubation, the fluorescence was determined at 540 nm and 590 nm, respectively, using a microplate reader (Tecan). The experiment was independently repeated three times.

### Membrane integrity study

Membrane integrity of HepG2 cells was assessed by evaluating the amount of LDH leaking out from the cells in accordance with the manufacturer's instructions (CytoTox96 Non-Radioactive Cytotoxicity Assay). The LDH assay worked on the principle that cytosolic enzyme LDH is released from the damaged cellular membrane. As such, quantitative analysis to track the progress of polymers-induced cytotoxicity was possible by measuring the activity of LDH in the supernatant of the cell cultures. Briefly, the cells were subjected to **H1**, **P2** and **P5** polymers of varying concentrations for 1 h and 2 h. Each cell-free supernatant (50 μL per well) was transferred into a 96-well plate before adding in 50 μL of LDH-assay reaction mixture. Lysis solution (10 μL) was then added to the untreated cells to generate a maximum LDH release control. After 1 h and 2 h of incubation under room temperature, the optical density of the color generated was measured at a wavelength of 490 nm using a Tecan microplate reader. The percentage cytotoxicity can be calculated as follows:

Percentage Cytotoxicity = 100 × (Experimental LDH Release (OD_490_)/Maximum LDH Release (OD_490_))

### Cellular uptake analysis

In an attempt to further determine the cellular uptake of the polymers, **H1** and **P5** were first labelled with AlexaFluoro 488 dye (Thermo Fisher Scientific Inc., Waltham, U.S.A.) according to a previously reported protocol, and the experimental procedures and reaction scheme (Scheme S2) can be found in the supplementary information 44. HepG2 cells (density: 5000 cells per well) were allowed to adhere and grow overnight in a four-well cover slip chamber (Lab-Tek II). After that, the spent medium was discarded and supplied with equivalent volume of medium containing AlexaFluoro 488-tagged **H1** and **P5** polymers at IC_50_ (polymer concentration which leads to 50% inhibition of cancer cell growth) and 2 × IC_50_ concentrations. After 30 min or 1 h of incubation, the cells were rinsed thrice with PBS to get rid of the remaining free polymer. Fixation of the cells was done by treating the cells with 4% formaldehyde (Sigma-Aldrich) for 15 min at 37 ºC and washed with PBS for 3 times prior to staining with Image-IT^TM^ Live Plasma Membrane and Nuclear Labelling Kit (Invitrogen, Singapore) according to the manufacturer's instructions. The treated cells were monitored using a confocal microscope (Zeiss LSM710 oil immersed 40× objective lens).

### Apoptosis study of cells by flow cytometry

Using a 12-well plate, HepG2 cells (density: 100,000 cells per well) were seeded and allowed to adhere and proliferate overnight prior to being treated with **H1**, **P2** or **P5** for 30 min and 60 min at different concentrations, respectively. Paclitaxel (2.8 μg/mL) and 0.01% Triton X-100 treated HepG2 cells served as controls for apoptosis and necrosis, respectively. Subsequently, all cells were collated into a 2 mL Eppendorf tube and centrifuged (1500 rpm, 5 min) to remove the supernatant. Annexin Binding Buffer was added to resuspend the cell pellets prior to centrifugation. Finally, and the cells were stained using propidium iodide (PI) and AlexaFluoro 488 dye, and incubated for 15 min at 25 ºC. After incubation, 200 μL of Annexin Binding buffer was added to each tube before placing the samples on ice. The stained cells were subjected to flow cytometry analysis (BD FACS LSR II). Three independent experiments were performed altogether.

## Results and Discussions

### Synthesis of guanidinium homopolymers (H1) and copolymers (P1-10)

Cationic and hydrophobic monomers containing Boc-protected guanidinium (MTC-OBu-BocGua) or hydrophobic (MTC-OR) functional groups were prepared separately. Prior to the synthesis of guanidinium-functionalized polycarbonates, guanidinium-functionalized alcohol precursors were first synthesized using a synthetic route reported previously 35. Briefly, 1,3-bis(tert-butoxycarbonyl)-2-methyl-2-thiopseudourea was allowed to react with 4-amino-1-butanol readily under ambient conditions. The concomitant loss of MeSH as the by-product afforded boc-protected guanylated alcohol. Subsequently, the desired guanidinium-functionalized monomer was efficiently accessed through acid promoted cyclisation of bis-MPA and the boc-protected guanylated alcohol 42. On the other hand, the hydrophobic monomers **M1-M5** were readily synthesized from bis-MPA and the respective alcohol of interest, followed by cyclisation using ethyl chloroformate under nitrogen (Scheme 1) 45.

**Figure 2:**
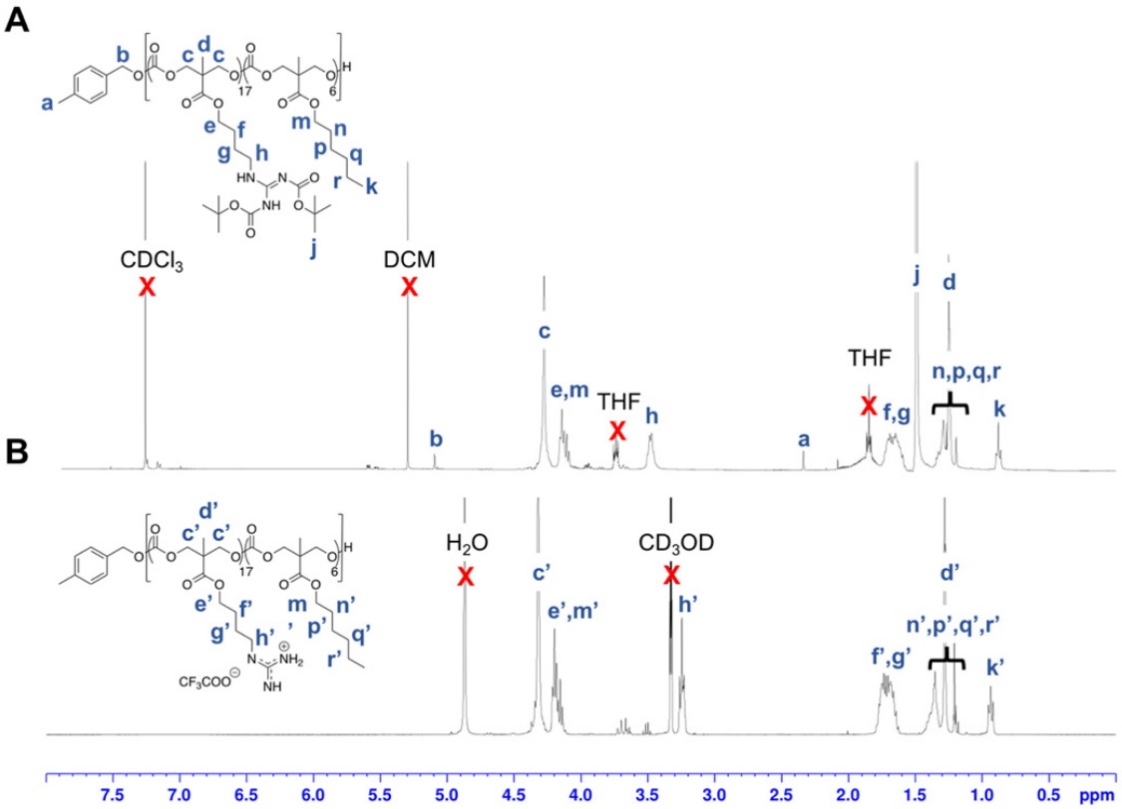
^1^H NMR spectra of **P5'** in (A) CDCl_3_ and deprotected **P5** in (B) CD_3_OD.

Polymerization was subsequently performed by the concurrent random copolymerization of MTC-OBu-BocGua and the respective MTC-OR with 4-methylbenzyl alcohol (4-MBA) as the initiator, and DBU and TU as co-catalysts to promote OROP. Subsequently, deprotection of the Boc-protected guanidinium with 10% TFA yielded the random copolymers **P1**-**P10** (Scheme 1). The controversy with regards to potential metal-caused cytotoxicity due to the presence of residual catalyst is circumvented due to the metal-free nature of the catalysts and low catalyst loading. Taking **P5** as an example, from the ^1^H-NMR spectra, quantitative comparisons between integral intensities of the peaks of -C*H*_2_N- protons of Gua at 3.47-3.42 (m, 33H) with that of the methyl protons of the hydrophobic monomer at 0.88 (t, J = 6.64 Hz, 17H) (Figure 2) provided an approximation of the copolymer's compositions. ^1^H-NMR characterization of the resulting polymer elucidated that there were 17 MTC-OBu-BocGua and 6 MTC-OHex units presented in the copolymer, with an average DP obtained to be in congruence with that predicted from initial monomer/initiator feed ratio. Furthermore, SEC results revealed that all the polymers have a narrow molecular weight distribution with Ð = 1.12-1.27 prior to deprotection (Table 1 and Figure S3). After deprotection using TFA, the cationic guanidinium-functionalized random copolymers were obtained as solids after freeze-drying. Taken together, functional and well-defined polycarbonates with predicable molecular weights and narrow molecular weight distribution could be easily attained using the expedient and highly controlled OROP method.

Amphiphilic balance in a polymer has a major influence in its interaction with the membrane and its internalization into the mammalian cells 46. Any alterations in the structures of the copolymers, either by varying the length of the alkyl chain of the hydrophobic side chains and/or tuning the composition of the polymers, could result in a significant change in the amphiphilicity of the resultant polymers. As such, a series of dansyl initiated polymers was synthesized following the protocol for the synthesis of the polymers to study their partition coefficient (log *P*) (Scheme S1).

### *In vitro* anticancer activity of the polymers

**Figure 3:**
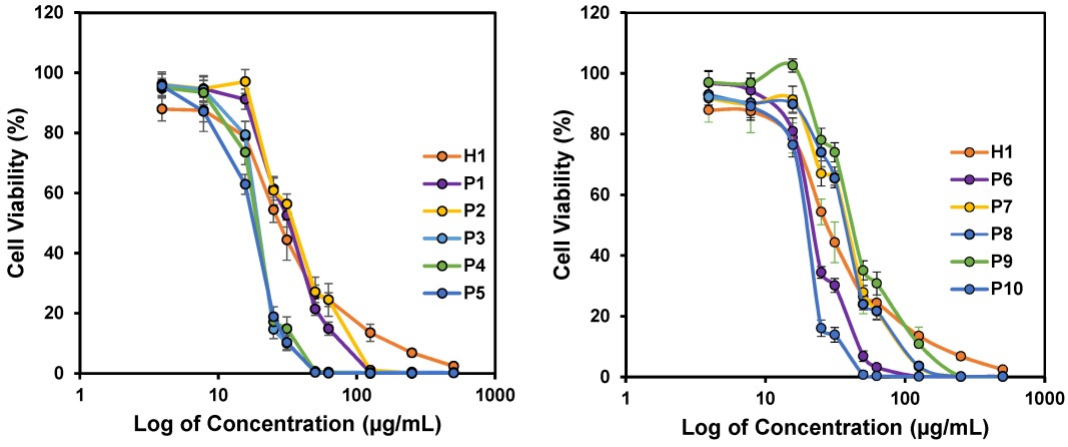
*In vitro* anticancer activity of polymers against HepG2 cancer cell lines after 24 h incubation. Experiments were done in triplicates (n = 3) and results were expressed as the mean ± standard deviation shown by the error bars (mean ± SD).

The success of our design approach was first evaluated by investigating the anticancer activity of the copolymers against HepG2 cancer cells. The anticancer efficiency was highly dependent on the concentration of the polymers used, and achieved 100% killing in the culture at high polymer concentrations (Figure 3). The IC_50_ values after 24 h treatment varied from 18 to 40 µg/mL, depending on the polymer composition (Table 2). All the polymers demonstrated excellent anticancer activity where the IC_50_ values were all below CMCs of the polymers (Table 2). This proved that the polymers were able to exert their anticancer activity even without the need for the formation of micelles. Thus, the hydrophobic side chains will be sufficiently revealed, increasing the activity of the polymers against HepG2 cancer cells. **H1**, **P2** and **P5** were further evaluated against MCF-7 and SW480 cell lines. Consistent with IC_50_ values against HepG2 cells, these three polymers showed considerable killing efficacy towards the respective tested cell lines. Notably, increasing the hydrophobicity from **P2** to **P5** enhanced the anticancer efficacy with reduced IC_50_ values across all cell lines tested (Figure 3 and Figure S1). These results demonstrated a broad spectrum of anticancer activity regardless of the identity of the hydrophobic groups.

The cytotoxicity of polymers **H1**, **P2** and **P5** was evaluated against the healthy human kidney HK2 cell line (Figure S2). Like many anticancer chemotherapeutics 47, 48 these polymers showed toxicity towards the healthy cells. Similar to HepG2, MCF-7 and SW480 cancer cell lines, the IC_50_ values of the polymers against HK2 also decreased as the hydrophobicity of the polymer increased from **H1** to **P2** to **P5**. These results suggest that the high hydrophobicity of polymers led to undesired cytotoxicity against the healthy human cell line that is likely due to non-specific hydrophobic binding to the healthy cell membrane. For future *in vivo* applications, the anticancer polymers may be formulated into nanoparticles that can transport them specifically to tumor tissues to mitigate their toxicity towards healthy tissues.

Hydrophobicity balance is regarded to be an essential aspect in anticancer activity and hence, the cationic copolymers were designed by introducing varying units of hydrophobic comonomer. The corresponding relationship between the overall hydrophobicity of these polymers on their anticancer activity can be further explained by determining the hydrophobic/hydrophilic balance through the investigation of the water-octanol partition coefficient (log *P*) of the polymers. A modified hydroxyl-terminated dansyl molecule served as the initiator to generate a series of highly fluorescent polymers (Scheme S1). A log *P* value below 0 is regarded to be hydrophilic, whereas a log *P* value above 0 is considered to be hydrophobic. While it is highly conventional to use log *P* values to examine the structure-activity relationship for small molecules, such approach is anticipated to be fairly pertinent for the quantitative measure of the polymers' overall hydrophobicity.

**Figure 4:**
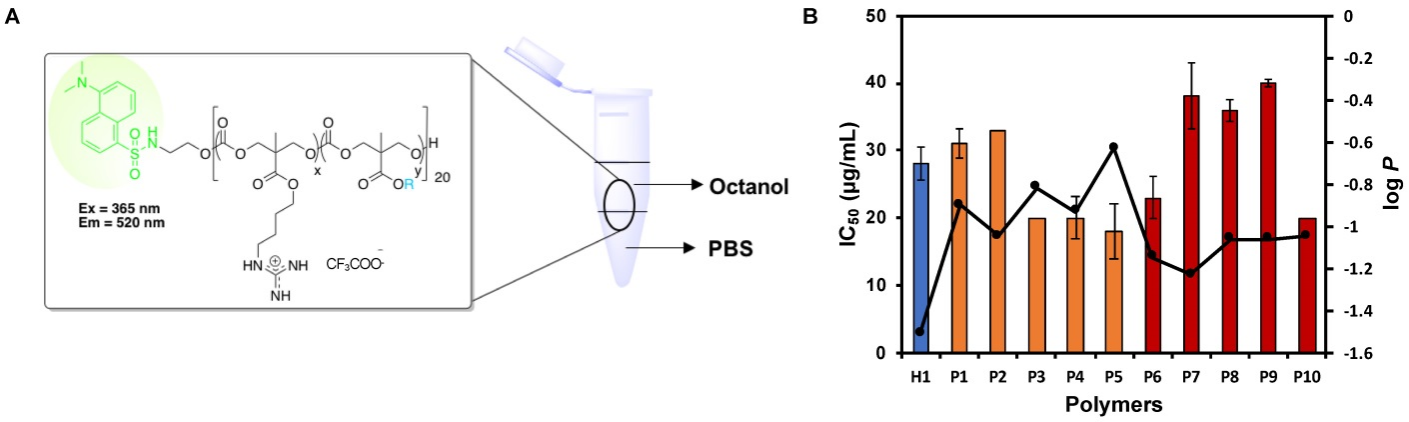
Effect of log P of polymers on anticancer activity (IC_50_). (A) Schematic for octanol-water partition coefficient measurement of the dansyl-labelled polymers. (B) Correlation between log P and IC_50_ of the polymers. The composition of the polymers was shown to have an impact on their hydrophobicity and thus, their corresponding anticancer activity. Experiments were done in triplicates (n = 3) and results were expressed as the mean ± standard deviation shown by the error bars (mean ± SD).

The log *P* values of the representative dansyl-labelled polymers are all below 0, suggesting their predominantly hydrophilic nature. Nonetheless, the log *P* values correlated well with its corresponding anticancer activity especially for the polymers **P2**-**P5** with the greater hydrophobic monomer content of 22-25% (Table 2, Figure 4). The longer the alky chain length, the more hydrophobic the polymer, and they are concomitantly more potent toward the cancer cells. As the chain length of the alkyl substituent of the hydrophobic homopolymers increased, i.e., from ethyl to butyl (*i*-butyl) and Hex groups, their respective log *P* increased from -1.05 to -0.82 (-0.93) and -0.63 for **P2**-**P5**, respectively, suggesting the increased overall hydrophobicity of the polymers with the increased hydrophobic alkyl chain length. The polymers **P3-P5** with the higher log *P* values demonstrated greater anticancer activity as evidenced by lower IC_50_ values as compared to **P2** (Table 2). Kuroda et al. 49 previously demonstrated the snorkeling effect of the methacrylate random copolymers, whereby the elongated cationic side chains led to increased depth of polymer insertion in anionic bacterial cell membrane, resulting in higher antibacterial activity. Owning to the similarities between the bacteria and cancer cell membranes as described previously, we hypothesized that longer cationic side chains might encourage the polymers to insert themselves into cancer cell membrane and disrupt the membrane more effectively, thereby leading to stronger anticancer activity. **P3** and **P4** bear butyl and isobutyl side chains, respectively, exhibited similar hydrophobicity, and thus their anticancer activity was comparable.

Significant difference in log *P* value was not observed among the polymers **P8-P10** with the lower hydrophobic monomer content of 11%. It is hypothesized that the relatively lower hydrophobic monomer content in these polymers rendered them insensitive to any changes in the alkyl chain length and thus, their log *P* remained relatively similar despite the increased in the hydrophobic chain length. For the polymers **P7-P10**, the significant decrease in IC_50_ value was only observed when the hydrophobic alkyl chain length increased from ethyl to hexyl (Table 2). Although the overall hydrophobicity of **P8-P10** was similar (close log *P* values), the IC_50_ values of **P8** and **P9** were almost twice as much as that of **P10**. This is possibly due to stronger interaction of hexyl groups with the cell membrane.

In addition, it was observed that the polymers with a higher hydrophobic monomer content exhibited higher overall hydrophobicity with greater log *P* values (Table 2, **P2** *vs.* **P7**, **P3** *vs.* **P8**, **P4** *vs.* **P9**), leading to stronger anticancer activity. Despite having a higher number of hydrophobic units in the polymer **P1**, being significantly more hydrophobic than **P6**, **P1** exhibited a significantly higher IC_50_ value as compared to **P6**. **P1** contains a higher amount of the bulky hydrophobic group benzyl than **P6**, which might interact with proteins present in the cell culture medium (slight cloudiness observed) 50, thus masking its anticancer activity. Another possible reason is that **P6** has a higher number of cationic guanidinium groups, which might enhance interaction between the polymer and the cell membrane, leading to stronger anticancer activity. Although **P10** had a higher log *P* value than **P5**, both had comparable anticancer activity, indicating that the long hexyl chain could interact with cell membrane effectively even its content in the polymer was low.

The **H1** is a control cationic polymer that contains only cationic groups and thus, is relatively hydrophilic (Table 2). Despite being highly hydrophilic, **H1** exhibited excellent anticancer activity against HepG2 cells. This phenomenon could be understood by the high charge density of the polymer as it possessed a higher composition of the cationic guanidinium repeating units as compared to the rest of the polymers, which might facilitate the attraction of the polymer onto the surface of the cancer cells and thus enhanced anticancer activity. These results indicated the importance of hydrophobicity/hydrophilicity (cationic charge) balance.

**Figure 5:**
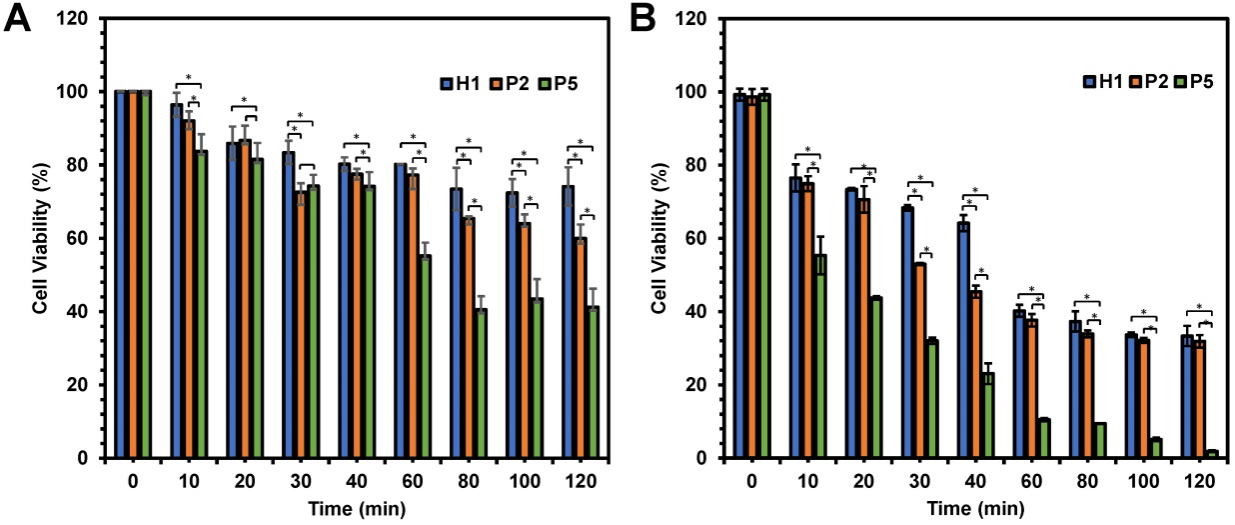
Viability of HepG2 cells as a function of time after being treated with **H1**,** P2** and **P5** at (A) 20 µg/mL and (B) 40 µg/mL. The killing kinetics of the cancer cells was dependent largely on the hydrophobicity and concentration of the polymers. The experiment was performed with 3 independent replicates, and the results were expressed as mean % cell viability ± standard deviation shown by the error bars. Student t-test was used to calculate significance. *p < 0.05.

In an attempt to determine the influence of the hydrophobicity has on the killing kinetics of the polymers, anticancer activity of **P2** and **P5** polymers was evaluated as a function of time in comparison with **H1**. As the hydrophobicity of the polymers increased from **H1** to **P2** to **P5**, the viability of HepG2 cells decreased significantly from 74% to 60% and 41% respectively (p < 0.05), after 120 min of incubation at polymer concentration of 20 µg/mL (Figure 5A). A similar trend was also seen at 80 and 100 min (p < 0.05). The most hydrophobic **P5** exhibited the most rapid killing, resulting in close to 60% cell death within 80 min. On the other hand, the relatively hydrophilic **H1** showed a much slower killing kinetics, causing only about 20% of cell death even after 120 min of incubation. In addition, the killing kinetics of cancer cells was also influenced by concentration of the polymers used. As shown in Figure 5B, these polymers displayed faster killing kinetics, with close to 100% of the cells being killed within 120 min upon treatment with 40 µg/mL of **P5**. At the higher concentration, **P5** also showed the fastest killing of cancer cells (p < 0.05). Gathered together, such results highlighted that the rate of killing kinetics of the cancer cells was dependent on the hydrophobicity and concentration of the polymers used.

### Degradability of the polymers

**Figure 6:**
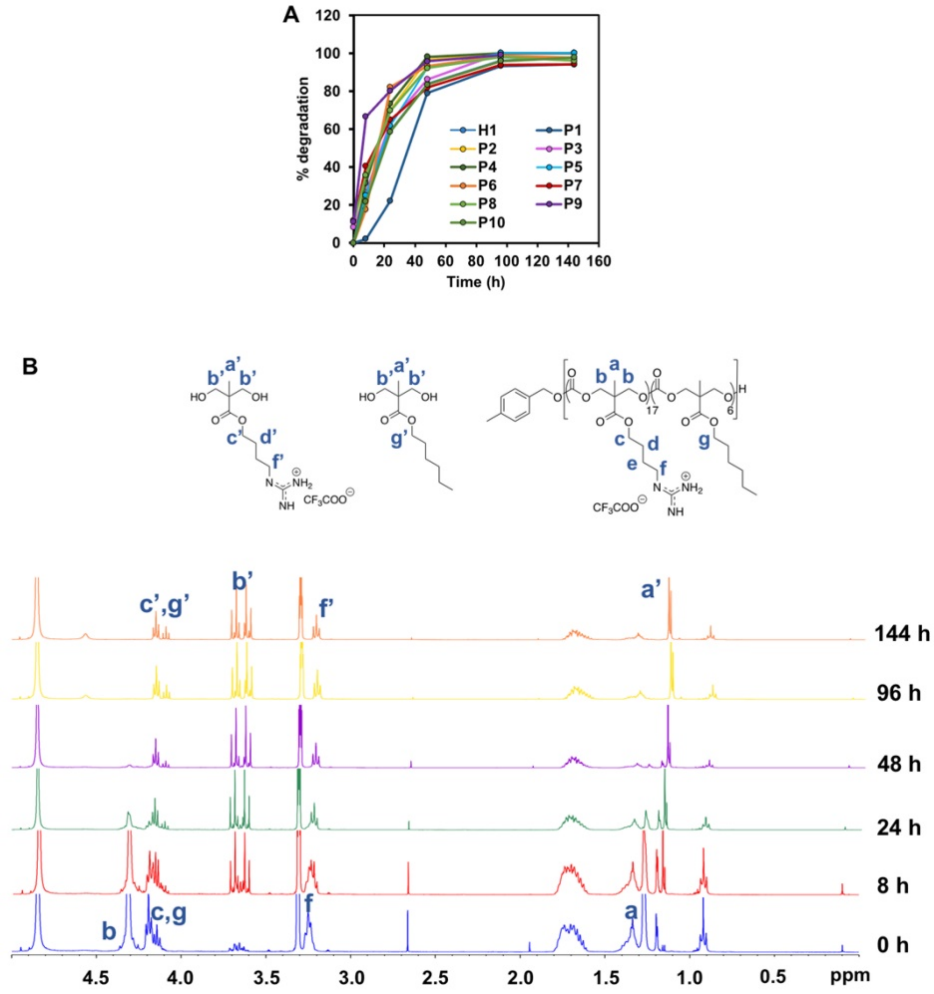
** (A)** Degradation time course of **P5** in PBS at 37 °C monitored by (B) ^1^H-NMR (400 MHz, CD_3_OD).

Degradability is a key characteristic of these amphiphilic polycarbonates and understanding their rate of degradation and behavior is of paramount for predicting the materials lifetime. Cationic polycarbonates equipped with guanidinium groups reported in our previous study demonstrated that most of the homopolymers were able to degrade between 3 and 6 days 44. Hydrophobicity plays an essential role in controlling the rate of degradation likely as a result of limited water penetration to the carbonate and ester bonds to undergo hydrolytic cleavage for the more hydrophobic polymers 51, 52. Therefore, in order to determine how hydrophobicity can affect the rate of degradation of the copolymers reported in this study, all the copolymers were subjected to hydrolysis. Figure 6A represents the time course of ^1^H-NMR of **H1**, **P1-P10** in PBS at 37 °C. In contrast to expectation that the more hydrophobic the polymer is, the slower the rate of degradation, the hydrolysis of the copolymers proceeded similarly fast as compared to the degradation of the homopolymer. In fact, most of the polymers were shown to have similar degradation rate irrespective of the hydrophobicity, and complete degradation occurred between 4 and 6 days. Figure 6B represents the time course degradation ^1^H-NMR of **P5** polymer. In general, hydrolysis took place gradually after 8 h, with several signals (a' to g') appeared in the NMR spectrum. **P1** degraded relatively slower in the first 2 days. The concentration of the polymers used in the degradation study was above their CMC values. **P1** contains a relatively high number of rigid benzene rings, which can form π-π stacking in the core of the micelles that were self-assembled from the polymer. This might lead to a rigid core and thus reduced accessibility of water molecules, slowing degradation rate. Nonetheless, the complete degradation of **P1** was seen in 6 days. Despite the fast hydrolytic rate of these polymers, they were able to kill the cancer cells effectively before they degrade substantially, as the copolymers were found to have a fast-killing kinetics with nearing 100% killing of the cancer cells within 2 h of incubation (Figure 5).

### Membrane-disruptive activity

The anticancer mechanism of the copolymers was further determined through the examination of the effect of polymer treatment on HepG2 cancer cell membrane. Cell membrane damage induced by **H1**, **P2** and **P5** copolymers was analyzed by assessing the extent of LDH release from the cells with damaged membrane or the lysed cells, which is also a characteristic marker for cell death. LDH catalyzed the conversion of lactate to pyruvate through NAD^+^ reduction to NADH. Subsequently, the enzyme diaphorase capitalizes on the NADH present in the cell culture medium to reduce tetrazolium salt to a red formazan product. The amount of formazan formed is highly dependent on the level of LDH released, and hence signified the degree of cytotoxicity 53. Therefore, by measuring the level of LDH, we can assess the status of cells under a given condition.

**Figure 7:**
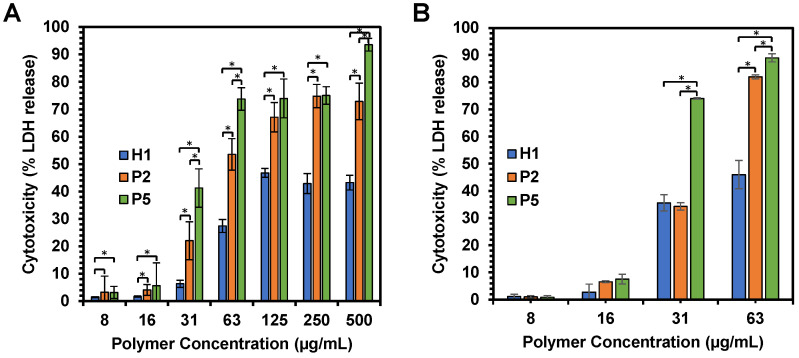
(A) Effects of **H1**, **P2** and **P5** on lactate dehydrogenase activity in HepG2 cancer cells after 1 h and (B) 2 h of incubation. The cells were treated with the polymers of varying concentrations and the LDH was measured by changes in optical densities. Results were expressed as the mean ± standard deviation shown by the error bars (n = 6). Student t-test was used to calculate significance. *p < 0.05.

We first examined membrane integrity of the cells treated with **H1**, **P2** and **P5** across a range of concentrations (7.81 µg/mL-500 µg/mL) over 1 h and 2 h exposure (Figure 7A,B). Under these conditions, the results suggest that cell membrane damage was dose-dependent, which increased with an increased polymer concentration (Figure 7A). Such results were consistent with cell viability, whereby an increase in the concentration of **H1**, **P2** and **P5** caused higher cytotoxicity. In addition, the amount of LDH release is well correlated with the hydrophobicity of the polymers used, with a greater amount of LDH being released as the hydrophobicity of the polymers increased in the order of **P5** > **P2** > **H1** (Table 2) at polymer concentrations of 31, 63 and 500 µg/mL (p < 0.05). As compared to the control **H1, P2** and **P5** induced a greater extent of membrane disruption at most polymer concentrations tested (Figure 7A, p < 0.05). **P5** caused 40% LDH release from the cells at a low polymer concentration of 31 µg/mL, whereas the less hydrophobic **P2** caused about 20% LDH release at the same concentration, indicating that **P2** treatment led to less membrane damage (p < 0.05). **P5** induced close to 100% LDH release at 500 µg/mL, demonstrating that cell death was caused mainly by membrane lysis. In contrast, negligible LDH release was observed in HepG2 cells treated with **H1** at low concentrations**.** Even at 500 µg/mL,** H1** only caused 50% LDH release. The results indicated that **H1** killed cancer cells predominately by membrane translocation followed by precipitation of cytosolic biomolecules 44. Taken together, these findings highlighted the contribution of the hydrophobic groups in causing membrane leakage as the more hydrophobic polymers caused a greater degree of cell-membrane damage and subsequent greater cytotoxicity to the cancer cells.

### Exploration of anticancer mechanism by confocal microscopy and flow cytometry

**Figure 8:**
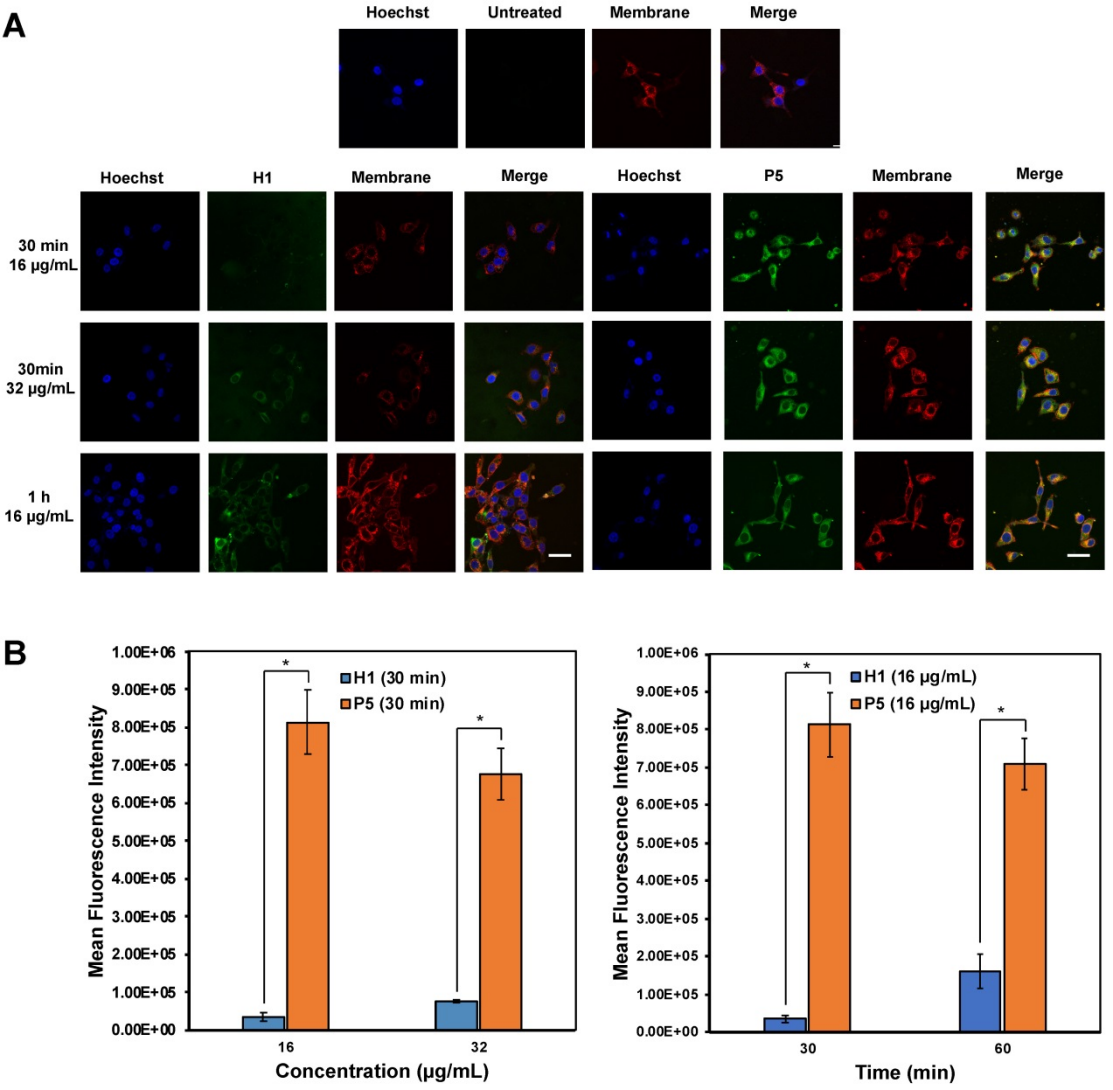
(A) Confocal microscopic images of HepG2 cells after treatment with **AF488-H1** and **AF488-P5** at 16 and 32 µg/mL for 30 min or 1 h. Hoechst (blue): nucleus; Green: AlexaFluor488-labelled **H1** and **P5** respectively; Red: cell membrane. The presence of the polymers in the cytosol even after a 30 min incubation time signified rapid membrane translocation activity of the polymer. Scale bar: 40 μm. (B) Mean fluorescence intensity of **H1** and **P5** at 16 μg/mL and 32 μg/mL of **H1** and **P5** at 30 min and 60 min. Results were expressed as the mean ± standard deviation shown by the error bars (n = 3). Student t-test was used to calculate significance. *p < 0.05.

Confocal imaging on the uptake of AF488-labelled **H1** and **P5** by HepG2 cells was first explored to determine the anticancer mechanism of the polymers (Figure 8A). It is recognized that guanidinium-rich anticancer peptides can translocate across various types of biological barriers 54. Bright green regions within the cells represent AF488-associated fluorescent emission produced by **AF488-H1** and **AF488-P5** polymers. Blue fluorescent dye stains cell nuclei, whereas the red fluorescent dye stains the cell membrane. Overlapping the fluorophore emissions from the same cell monolayer gave rise to the merged confocal images. As shown in Figure 8A, polymer molecules (green fluorescence) were seen on the cell membrane and inside the cells at 30 min and 1 h, demonstrating that the polymers were taken up rapidly by the cells even over a short incubation time. Particularly, the co-localization of the polymers and the cell membrane was evidenced by the presence of the yellow regions. In addition to denoting an effective cellular uptake process, the fluorescence patterns suggested the importance of the hydrophobic group to facilitate the cellular accumulation of the polymers. The images reflected a higher **P5** uptake by HepG2 cancer cells as compared with homopolymer **H1** over the same concentration and the same incubation time (Figure 8B). Therefore, these findings suggested the influence of the hexyl hydrophobic group in facilitating the internalization and accumulation of the polymer into the cell. The results obtained are consistent with the findings of cancer cell killing kinetics, where the more hydrophobic polymer **P5** killed cancer cells more rapidly than **H1** (Figure 5).

**Figure 9:**
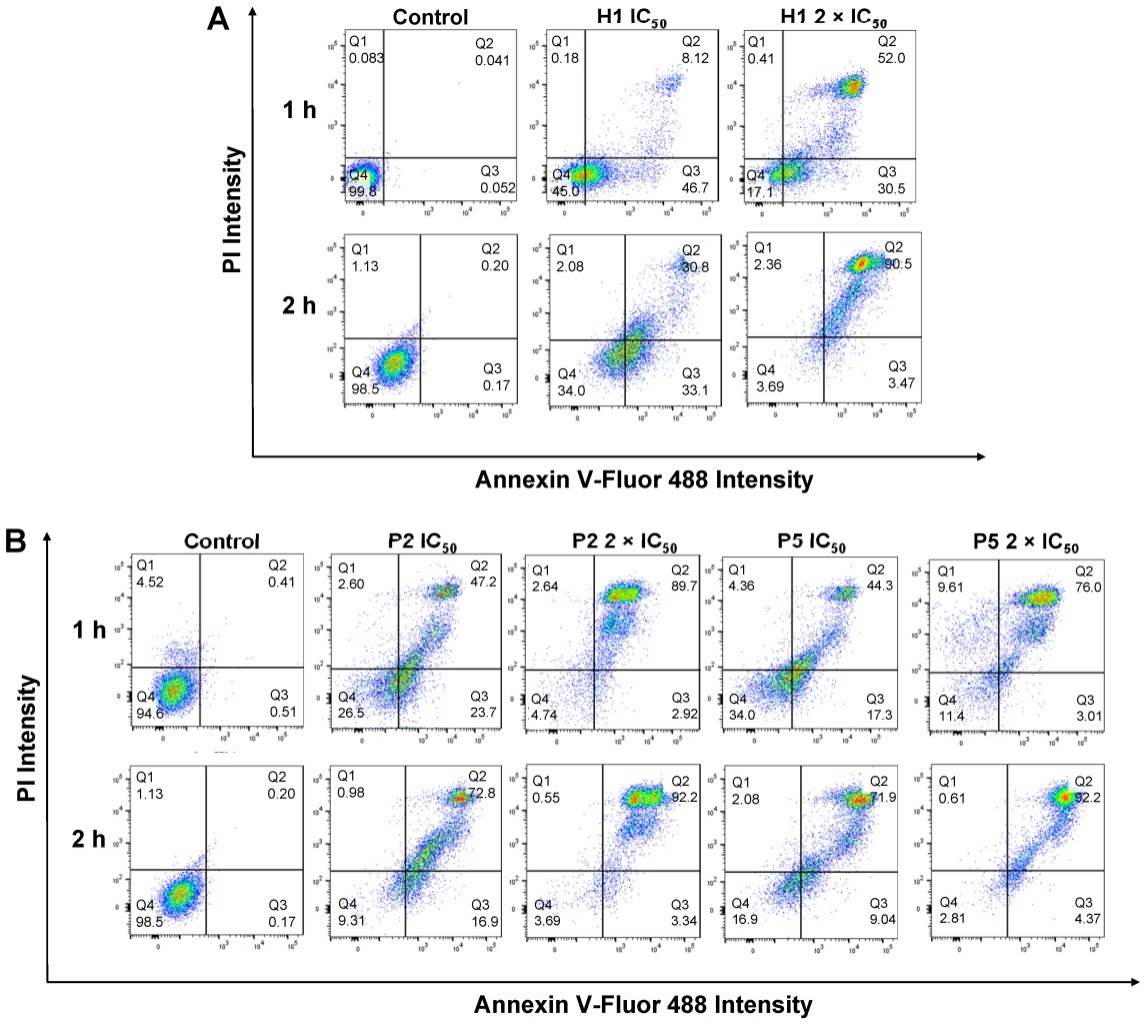
Apoptosis/necrosis of HepG2 cells was detected by PI/Annexin V staining after treatment with (A) **H1**, (B) **P2** and **P5** for 1 h and 2 h at IC_50_ and 2 × IC_50._ PI^low^/ Annexin V^low^: live cells; PI^low^/ Annexin V^high^: early apoptosis cells; PI^high^/ Annexin V^low^ or PI^high^/ Annexin V^high^: necrotic cells. Control: no polymer treatment. The data are representative of 3 replicates.

To understand if the polymers killed cancer cells based on apoptotic or necrotic mechanism, the cells were analyzed by flow cytometry after 1 h or 2 h of treatment with **H1, P2 or P5** at different concentrations (Figure 9A,B). To discriminate between necrosis and apoptosis, HepG2 cells treated with **P2**, **P5** and **H1** were stained by AF488-Annexin V and PI prior to flow cytometry analysis. Annexin V stains apoptotic cells, while PI penetrates the broken cell membrane and is readily internalized into the necrotic cells.

As seen from Figure 9B, the treatment of cancer cells with **P2** at IC_50_ (33 µg/mL) for 1 h led to a greater number of apoptotic cells and a comparable number of necrotic cells as compared to the control cells (apoptotic: 23.7% *vs.* 0.51%, PI^low^/Annexin V^high^; necrotic: 2.60% *vs.* 4.52%, Annexin V^low^/PI^high^), indicating apoptotic mechanism. This is in agreement with membrane disruption study, where only negligible LDH release was seen (Figure 7A). Extending the treatment time from 1 h to 2 h at the polymer's IC_50_ decreased the number of apoptotic cells (23.7% *vs.* 16.9%), while increasing the number of dead cells (49.8% *vs.* 73.8%, PI^high^/Annexin V^high^). Despite the significant surge in the percentage of dead cells, the amount of LDH released after 1 h and 2 h at the polymer's IC_50_ only increased slightly from 22% to 34% (Figure 7A,B), indicating that cell death occurred predominantly via apoptosis. On the other hand, increasing the polymer concentration to 2 × IC_50_ under the same incubation conditions caused the number of apoptotic cells to decrease significantly (2.92% and 3.34% for 1 h and 2 h of incubation, respectively *vs.* 23.7%), whereas most of the cells were dead (89.7% and 92.2% for 1 h and 2 h of incubation, respectively). At 2 × IC_50_, **P2** caused more than 50% (1 h incubation) and 80% (2 h incubation) LDH released, and it killed cancer cells possibly based on both apoptotic and necrotic mechanisms. Similar trends were also observed on cells treated with **P5** under the same conditions. The treatment of cancer cells with **P5** at IC_50_ (18 µg/mL) for 1 h led to a higher number of apoptotic cells and a comparable number of necrotic cells as compared to the control cells (apoptotic: 17.3% *vs.* 0.51%; necrotic: 4.36% *vs.* 4.52%), indicating apoptotic mechanism. Increasing the treatment time from 1 h to 2 h at IC_50_ led to a lower number of apoptotic cells (9.04% *vs.* 17.3%), and an increased number of dead cells (71.9% *vs.* 44.3%). Nevertheless, apoptosis remained the main mechanism of cell death as the amount of LDH released by the cancer cells remained negligible (Figure 7A,B). At 2 × IC_50_, which led to 40% LDH release (Figure 7A), 1 h of treatment with **P5** caused an increased number of necrotic cells and a reduced number of apoptotic cells as compared to the lower polymer concentration of IC_50_. When the treatment time was prolonged to 2 h, most of the cancer cells were dead (92.2% *vs.* 76.0%), with 72% LDH being released (Figure 7B). These findings showed that similar to **P2**, incubation of the cancer cells with **P5** for 1 h and 2 h at IC_50_ killed the cancer cells likely based on apoptosis, while incubation at 2 × IC_50_ killed the cancer cells likely based on both apoptosis and necrosis.

In the case of **H1** treatment at IC_50_ over a time course of 1 h, a much higher number of apoptotic cells (46.7%) were observed as compared to the control cells (apoptotic: 46.7% *vs.* 0.052%, PI^low^/Annexin V^high^). In contrast, a negligible percentage of the cells were found in the necrotic region (necrotic: 0.18% *vs.* 0.083%, PI^high^/Annexin V^low^), indicating apoptotic mechanism (Figure 9A). This is in agreement with membrane disruption study, where only negligible LDH release was seen in cells treated with **H1** (Figure 7A). On the other hand, **H1** treatment at IC_50_ over a time course of 2 h, a higher number of apoptotic cells (33.1%) were observed as compared to **P2** (16.9%) and **P5** (9.04%). At IC_50_, **H1** treatment for 2 h resulted in only 36% LDH release (Figure 7B), indicating that **H1** killed the cancer cells mainly by inducing apoptosis. While most of the cancer cells were dead after 2 h of treatment at 2 × IC_50_, **H1** only contributed to less than 50% LDH release (Figure 7B). These results demonstrated that in contrast to **P2** and **P5**, the anticancer mechanism of **H1** was independent on incubation time and polymer concentration: mainly apoptosis at both IC_50_ and 2 × IC_50_ for 1 h and 2 h of incubation. Apoptotic anticancer mechanism was also reported on peptides by others 55-57. Detailed studies into the mechanism of the polymers will be performed in the future.

## Conclusion

In this study, the molecular design of guanidinium-functionalized polycarbonates was successfully established as a synthetic mimic of anticancer peptides. These polymers are hydrophilic in nature, and exert anticancer activity at concentrations below their CMC. They completely degrade within 4-6 days in PBS at 37 °C. The anticancer activity of these copolymers could be fine-tuned by varying the compositions of their cationic and hydrophobic monomers, as well as varying the structures of the hydrophobic monomers. Binding of the polymers onto the lipid membrane is more pronounced with increased hydrophobicity, which in turn causes membrane disruption and ultimate cell death. Being the most hydrophobic polymer bearing the hexyl side chain, **P5** is the most effective against cancer cells as compared to the other polymers. Like most anticancer drugs, the polymers showed toxicity towards healthy cells. They may be formulated into nanoparticles for targeting tumor tissues to mitigate toxicity towards healthy tissues. The anticancer mechanism of the polymers **P2** and **P5** bearing the hydrophobic counterparts depends on concentration. At IC_50_, they induce mainly apoptosis, while they kill cancer cells *via* both apoptosis and necrosis at 2 × IC_50_. In contrast, **H1** functions based on apoptotic mechanism at both IC_50_ and 2 × IC_50_. These polymers can potentially be used as anticancer agents.

### Statistical analysis

Statistical analysis was carried out using Student's t-test. A value of *p* < 0.05 was considered to be statistically significant.

## Supplementary Material

Supplementary materials and figures.

## Figures and Tables

**Table 1:** Characteristics of cationic polycarbonates surveyed in this study

Polymer	R	Composition		Hydrophobicity Content (%)	Precursor (Boc-)^a^				After deprotection
		x	y		M_n_^b^ (g mol^-1^)	M_n_^c^ (g mol^-1^)	Ð_M_^C^	PDI^c^	M_n_^b^ (g mol^-1^)
H1		20	0	0	9.59E+03	6.18E+03	1.19	0.45±0.04	5.55E+03
P1	Bn	12	6	33	7.31E+03	9.84E+03	1.15	0.13±0.02	4.88E+03
P2	Et	14	4	22	7.50E+03	5.66E+03	1.18	0.23±0.02	4.67E+03
P3	Bu	15	5	25	8.31E+03	8.48E+03	1.27	0.24±0.02	5.27E+03
P4	*i*-Bu	13	4	24	7.14E+03	8.95E+03	1.22	0.22±0.02	4.51E+03
P5	Hex	17	6	26	9.33E+03	7.22E+03	1.20	0.29±0.07	5.90E+03
P6	Bn	19	3	14	9.87E+03	6.40E+03	1.12	0.41±0.03	6.03E+03
P7	Et	16	2	11	8.08E+03	6.24E+03	1.18	0.33±0.05	4.84E+03
P8	Bu	17	2	11	8.60E+03	6.35E+03	1.20	0.17±0.03	5.17E+03
P9	*i*-Bu	16	2	11	8.13E+03	6.54E+03	1.18	0.18±0.05	4.90E+03
P10	Hex	16	2	11	8.16E+03	6.17E+03	1.19	0.20±0.04	4.93E+03

^a^ Polymers with a Boc group; ^b^ Determined by ^1^H-NMR; ^c^ Determined by SEC (THF).

**Table 2:** Biological activities of cationic polycarbonates

Entry	Polymer	R	CMC (µg mL^-1^)	Size	Log *P*	IC_50_ ± SD (ppm)
1	H1		342.3	357.0±9.3	-1.51	28±6.1
2	P1	Bn	182.8	97.8±1.8	-0.90	31±2.5
3	P2	Et	261.0	285.2±6.2	-1.05	33±2.2
4	P3	Bu	210.2	229.0±1.4	-0.82	20±0.0
5	P4	*i-*Bu	204.7	161.6±2.2	-0.93	20±0.0
6	P5	Hex	89.6	231.6±4.2	-0.63	18±3.2
7	P6	Bn	271.2	205.7±12.3	-1.15	23±4.0
8	P7	Et	380.8	256.6±7.5	-1.23	38±3.1
9	P8	Bu	334.0	302.8±4.0	-1.06	36±4.9
10	P9	*i-*Bu	345.5	226.2±2.6	-1.06	40±1.6
11	P10	Hex	168.4	241.1±2.4	-1.05	20±0.6

## Abbreviations

ACN: acetonitrile
AcOH: acetic acid
AMP: antimicrobial peptides
BHT: butylated hydroxytoluene
bis-MPA: 2,2-bis(hydroxymethyl)propionic acid
Bn: benzyl
Bu: butyl
CDI: *N,N'*-carbonyldiimidazole
CaH_2_: calcium hydride
CDCl_3_: deuterated chloroform
CMC: critical micelle concentration
DBU: 1,8-diazabicyclo[5.4.0]undec-7-ene
DCC: dicyclohexylcarbodiimide
DCM: dichloromethane
DI: deionized water
DIPEA: N,N-diisopropylethylamine
DMAP: 4-(dimethylamino)pyridine
DMEM: Dulbecco's modified eagle medium
DMSO: dimethyl sulfoxide
EA: ethyl acetate
Et: ethyl
Gua: guanidinium
Hex: hexyl
HCl: hydrochloric acid
HDPs: host defence peptides
H-NMR: hydrogen-1 nuclear magnetic resonance
*i*Bu: isobutyl
LDH: lactate dehydrogenase
MDR: multidrug resistance
MeOD: deuterated methanol
MTC: trimethylene carbonate
NAD: nicotinamide adenine dinucleotide
NADH: nicotinamide adenine dinucleotide + hydrogen
NaHCO_3_: sodium bicarbonate
NaSO_4_: sodium sulfate
OD: optical density
OROP: organocatalytic ring-opening polymerization
PBS: phosphate-buffered saline
PI: propidium iodide
rpm: revolutions per minute
RPMI: Rosewell Park Memorial Institute medium
SEC: size exclusion column
TEA: triethylamine
TFA: trifluoroacetate acid
THF: tetrahydrofuran
TU: N-(3,5-trifluoromethyl)phenyl-N'-cyclohexylthiourea
4-MBA: 4-methylbenzyl alcohol
IC_50_: half maximal inhibition concentration
