# Supplementary material for: Elucidating the anticancer activities of guanidinium-functionalized amphiphilic random copolymers by varying the structure and composition in the hydrophobic monomer
Source: Theranostics. 2021 Aug 21;11(18):8977–92. doi: 10.7150/thno.60711 (PMC8419055; doi:10.7150/thno.60711)
Supplement: Supplementary file 1 — Supplementary materials and figures. [file thnov11p8977s1.pdf]

Supplementary Information

Elucidating the anticancer activities of guanidinium-functionalized amphiphilic random copolymers by varying the structures and compositions of the hydrophobic monomer

Joyce Tay[†, ‡], Yanli Zhao[‡], James L. Hedrick[⊥], Yi Yan Yang[†, *]

[†] Institute of Bioengineering and Bioimaging, The Nanos, 31 Biopolis Way, Singapore 138669, Singapore

[‡] Division of Chemistry and Biological Chemistry, School of Physical and Mathematical Sciences, Nanyang Technological University, 21 Nanyang Link, Singapore 637371, Singapore

[⊥] IBM Almaden Research Center, 650 Harry Road, San Jose, California 95120, United States

ORCID: 0000-0003-3802-9901 (Joyce Tay), 0000-0002-9231-8360 (Yanli Zhao) 0000-0002-3621-9747 (James Hedrick), 0000-0002-1871-5448 (Yi Yan Yang)

[*] Corresponding author at Institute of Bioengineering and Bioimaging, The Nanos, 31 Biopolis Way, Singapore 138669, Singapore (Y.Y. Yang)

E-mail address: yyyang@ibb.a-star.edu.sg

Synthesis of dansyl-conjugated polycarbonates

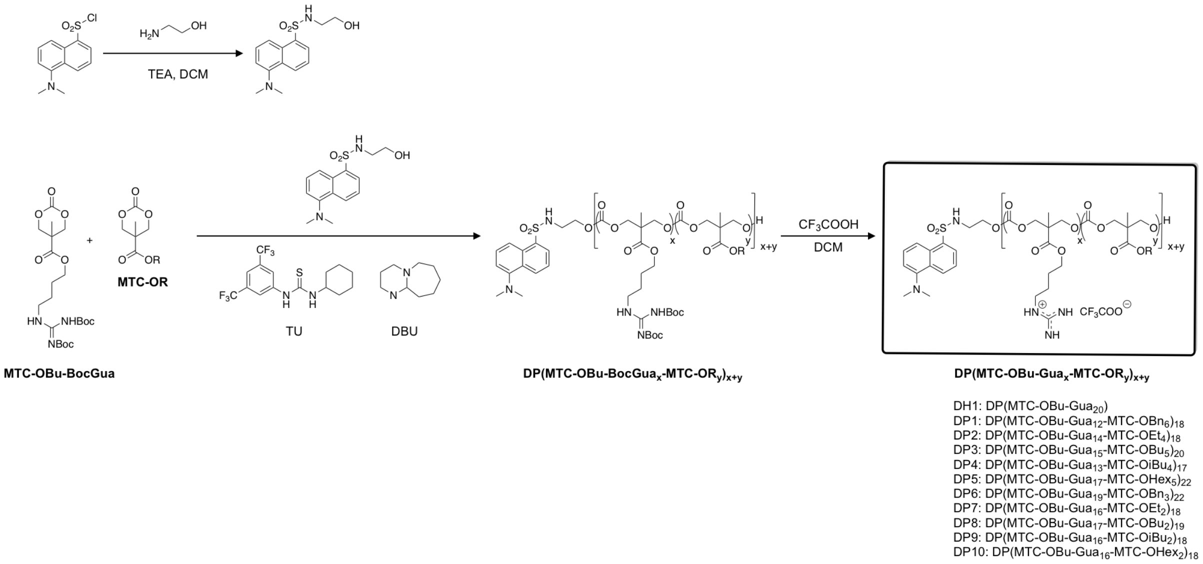

Scheme S1. General synthetic procedures and chemical structure of dansyl alcohol initiator and dansyl-conjugated polycarbonates.

Experimental

Synthesis of Boc-protected guanylated alcohol precursor (HO-Bu-BocGua)

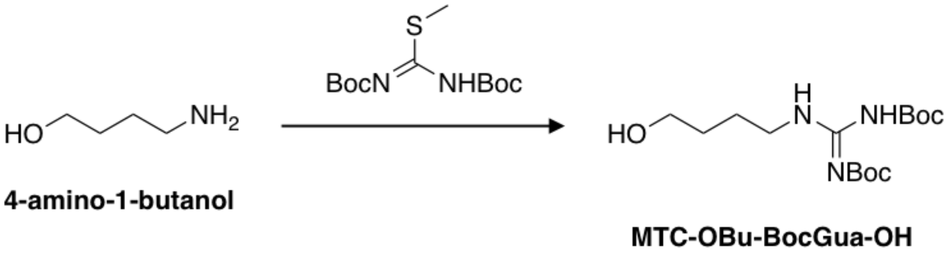

Boc-protected guanylated alcohol precursor was synthesized according to previously published protocols [1]. Briefly, 1,3-bis(tert-butoxycarbonyl)-2-methyl-2-thiopseudourea (3.30 g, 11.6 mmol, 1.0 equiv) was added into a solution of 4-amino-butanolamine (1.25 mL, 2.0 equiv) and N,N-diisopropylethylamine (DIPEA) (6.0 ml, 34.4 mmol, 3.0 equiv) in 20 mL of dry CH_2Cl_2 (DCM). The mixture was allowed to stir overnight at room temperature. Subsequently, the DCM was removed, and flash column chromatography was carried out to purify the crude product to yield Boc-protected guanylated alcohol (HO-Bu-BocGua) as a white solid (3.20 g, 90% yield). ^{1}H NMR (400 MHz, $CDCl_3$, 22 °C): δ = 11.48 (s, 1H, Ph –N*H*), 8.46 (S, 1H, N*H*),

3.70 (t, J = 6.1 Hz, 2H, –CH_2–), 3.50-3.45 (m, 2H, C$H_a$$H_b$ and C$H_a$$H_b$), 1.83-1.60 (m, 4H, -C$H_2$-), 1.50 (d, J = 2.68 Hz, 19H, Boc -CH_3).

Synthesis of 5-methyl-2-oxo-1,3-dioxane-5-carboxylic acid (MTC-OH)

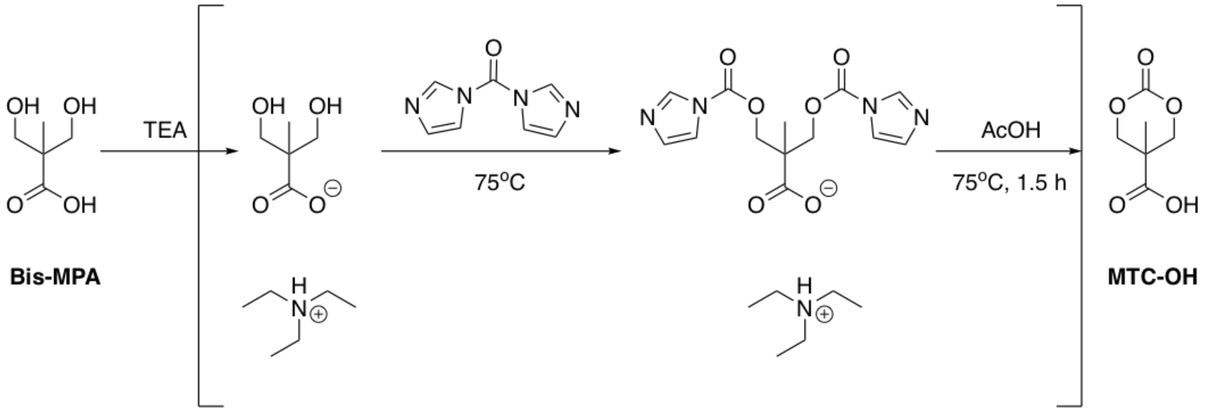

A mixture of bis-MPA (10.0g, 1 equiv), ethyl acetate (EA) (250 mL) and triethylamine (TEA) (10.4 mL, 1 equiv) was set aside to react at room temperature. Subsequently, carbonyldiimidazole (CDI) (24.2 g, 2 equiv) was added in two portions to the above mixture with 5 min interval and allowed to stir for 5 min prior to the addition of AcOH (17.1 mL, 4 equiv). The pot of solution was subjected to heating at 75 °C for 1.5 h. After that, cold acetone was added after removing the solvent via rotavap. Half of the swelled amberlyst 15 beads in cold ACN were added to the flask and left standing for 10 min before decanting into a column filled with beads. Acetone (1 L) was used to pass through the column to remove all traces of MTC-OH. The filtered solution was then filtered through a carbon filter before removing the solvent in vacuo. The resulting residue was dissolved in minimal EA and toluene (50 mL) was added to remove acetic acid [2]. The product was filtered and rinsed with minimal diethyl ether to afford **MTC-OH** as a white crystalline solid (10.3 g, 80%). ^{1}H NMR (400 MHz, DMSO, 22 °C): δ = 13.34 (bs, 1H, COO*H*), 4.53 (d, J = 10.2 Hz, 2H, C$H_a$$H_b$ and C$H_a$$H_b$), 4.31 (d, J = 9.8 Hz, 2H, C$H_a$$H_b$ and C$H_a$$H_b$), 1.16 (s, 3H, –C$H_3$).

Synthesis of MTC-OBu-BocGua monomers

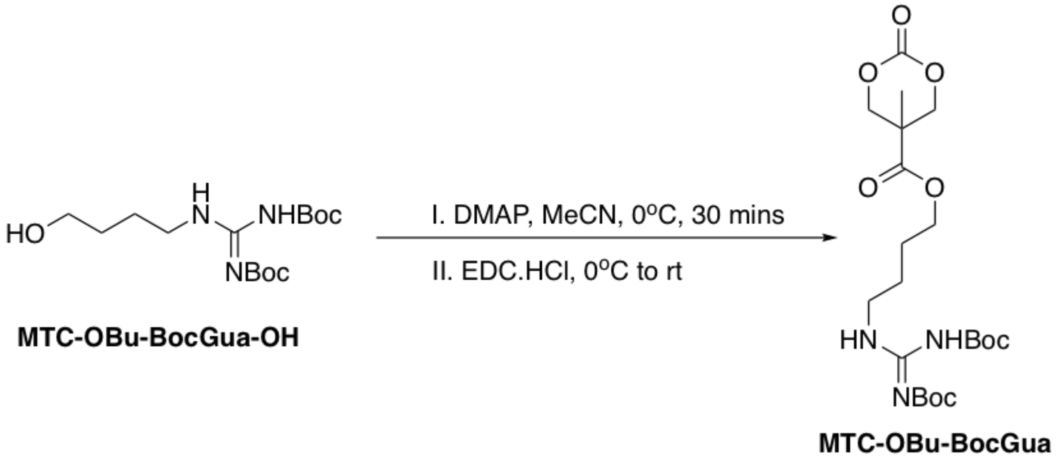

Cold ACN (250 mL) was used to dissolve the mixture containing MTC-OH (3.08 g, 19.3 mmol), HO-Bu-BocGua (8.60 g, 3.02 mmol) and DMAP (0.30 g, 2.5 mmol). The reaction mixture was cooled inside the ice bath to 0°C before EDC.HCl was added. The reaction mixture was stirred overnight under room temperature. Subsequently, ACN was removed completely, yielding a yellow oil. The oil was dissolved in DCM and subsequently extracted with water (500 mL), followed by 1N HCl (2 × 500 mL), saturated $NaHCO_3$ (2 × 500 mL) and lastly with brine (200 mL). The resulting organic layer was dried over $NaSO_4$ and the volatile was removed under high pressure. The resulting product was dissolved in a small amount of EA under sonication and precipitated out in hexane to afford a white solid (7.2 g, 74%). ^{1}H NMR (400 MHz, DMSO, 22 °C): δ = 11.49 (s, 1H, N*H*), 8.34 (s, 1H, N*H*), 1.76-1.62 (m, 6H, –CH_2- and water), 1.50-1.49 (m, 18H, Boc –CH_3), 1.35 (s, 3H, -CH_3).

Synthesis of hydrophobic monomers M1’-M5’

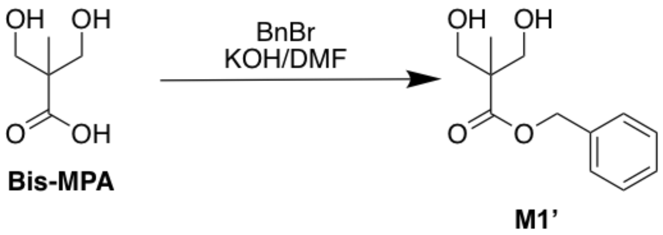

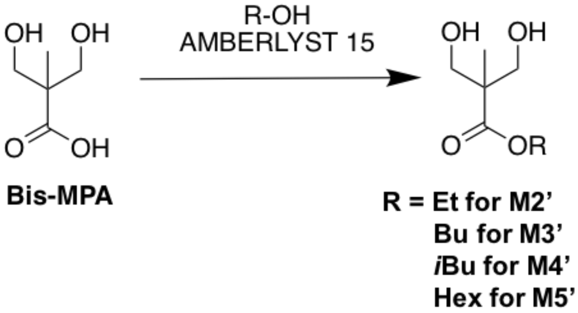

The synthesis of the hydrophobic monomers was modified from previously published protocols [3, 4]. The synthesis of **M1’ and M2’** are given as a representative example. A solution of bis-MPA (45 g, 0.336 mol) and KOH (21.5 g, 0.383 mol) in DMF (250 mL) was stirred at 100 °C for 1 h till all were dissolved. Following that, benzyl bromide (48.0 mL, 0.404 mol) was added and stirred for 24 h at 100 °C. Flash column chromatography was carried out to purify the resulting crude mixture to afford **M1’** as a white crystalline product (40.1 g, 53%). ^{1}H NMR (400 MHz, $CDCl_3$, 22 °C): δ = 7.39 – 7.30 (m, 5H, Ph –C*H*), 5.20 (s, 2H, –CH_2–), 3.92 (d, J = 11.2 Hz, 2H, -CH_aH$_b$ and -CH$_a$$H_b$), 3.72 (d, J = 11.3 Hz, 2H, -CH_aH$_b$ and -CH$_a$$H_b$), 3.10 (bs, 2H, O*H*), 1.08 (s, 3H, –CH_3).

For the synthesis of **M2’**, bis-MAP (22.1 g, 0.165 mmol) and amberlyst 15 beads (6.8 g) were charged into ethanol (150 mL) and reflux overnight. Subsequently, the beads were filtered off and the resulting filtrate was purified by flash column chromatography to yield **MPA-OEt (M2’)** (21.1 g, 79% yield). ^{1}H-NMR (400 MHz, $CDCl_3$, 22 °C: δ = 4.18 (q, J = 7.1 Hz, 2H, -OCH_2), 3.19 (bs, 2H, -O*H*), 3.86-3.65 (m, 4H, -CH_aH$_b$ and -CH$_a$$H_b$), 1.26 (t, J=7.14 Hz, 3H, methyl -CH_3), 1.04 (s, 3H, terminal -CH_3).

MPA-OBu (M3’): (26.1 g, 83% yield). ^{1}H-NMR (400 MHz, $CDCl_3$, 22 °C: δ 4.13 (t, J = 6.6 Hz, 2H, -OCH_2), δ 3.85 (m, 2H, -CH_aH$_b$ and -CH$_a$$H_b$), δ 3.67 (m, 2H, -C$H_aH_b$ and -

CH_aH_b), δ 1.62 (m, 2H, $-OCH_2CH_2$), δ 1.37 (m, 2H, $-CH_2CH_2CH_3$), δ 1.05 (s, 3H, $-CH_3$), δ 0.91 (t, J = 7.4 Hz, 3H, terminal $-CH_3$).

MPA-O*i*Bu (M4'): (21.7 g, 83% yield). ^{1}H-NMR (400 MHz, $CDCl_3$, 22 °C: δ 3.89 (d, J = 6.6 Hz, 2H, $-OCH_2$), δ 3.85-3.82 (m, 2H, $-CH_aH_b$ and $-CH_aH_b$), δ 3.67-3.64 (m, 2H, $-CH_aH_b$ and $-CH_aH_b$), δ 1.93 (septet, J = 6.7 Hz, 1H, $-CH(CH_3)_2$), δ 1.05 (s, 3H, $-CH_3$), δ 0.91 (d, J = 6.7 Hz, 6H, $-CH(CH_3)_2$).

MPA-OHex (M5'): (29.5 g, 82% yield). ^{1}H-NMR (400 MHz, $CDCl_3$, 22 °C: δ 4.13 (t, J = 6.7 Hz, 2H, $-OCH_2-$), δ 3.88 (d, J = 11.2 Hz, 2H, $-CH_2-$), δ 1.64 (quintet, J = 7.1 Hz, 2H, -OCH2CH_2), δ 1.37-1.21 (m, 6H, $-CH_2CH_2CH_2$), δ 1.04 (s, 3H, $-CH_3$), δ 0.87 (t, J = 6.9 Hz, 3H, terminal $-CH_3$).

Synthesis of cyclized hydrophobic monomers M1-M5

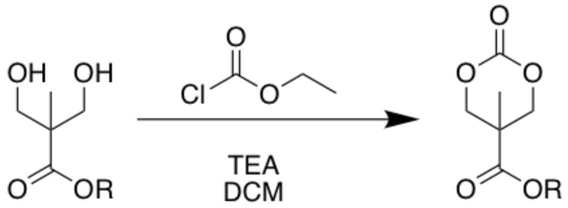

The synthesis of the hydrophobic monomers was modified from previously published protocol [3, 5]. The synthesis of **M1** is given as a representative example. Anhydrous THF (100 mL) was used to dissolve **M1'** (39.4 g, 0.176 mol) and the solution was cooled to 0 °C before the introduction of ethyl chloroformate (41.9 mL, 0.440 mol) under an inert flow of nitrogen. After that, the mixture of TEA (61.8 mL, 0.443 mol) in THF (100 mL) was added dropwise and the resulting solution was left to stir overnight. Once fully reacted, the precipitate formed was filtered off, and the filtrate concentrated to obtain the crude product as a yellow oil. The yellow oil was crystalized in cold Et_2O to yield a white solid. Following that, the white solid was re-dissolved in DCM, before being extracted with DI water (3 × 100 mL). The organic layer was allowed to dry over anhydrous Na_2SO_4. Finally, the solvent was removed in vacuo to yield **M1'** in the form of a white solid (39.3 g, 89% yield). ^{1}H NMR (400 MHz, $CDCl_3$, 22 °C): δ = 7.41 – 7.32 (m, 5H, Ph $–CH$), 5.21 (s, 2H, $–CH_2$), 4.71 (d, J = 10.9 Hz, 2H, $-CH_aH_b$ and $-CH_aH_b$), 4.21 (d, J = 10.9 Hz, 2H, $-CH_aH_b$ and $-CH_aH_b$), 1.33 (s, 3H, terminal $–CH_3$).

MTC-OEt (M2): (2.5 g, 72% yield)^{1}H-NMR (400 MHz, $CDCl_3$, 22 °C: δ = 4.69 (d, J = 10.9 Hz, 2H, -OCH_2), 4.29-4.18 (m, 4H, -C$H_a$$H_b$ and -C$H_a$$H_b$), 1.33-1.28 (m, 6H, methyl -CH_3 and alkyl terminal -CH_3).

MTC-OBu (M3): (2.3 g, 66% yield). ^{1}H-NMR (400 MHz, $CDCl_3$, 22 °C: δ = 4.63 (d, J = 10.9 Hz, 2H, -OCH_2), δ 4.18-4.13 (m, 4H, -C$H_a$$H_b$ and -C$H_a$$H_b$), δ 1.63-1.56 (m, 2H, -OCH_2-), δ 1.37-1.28 (m, 2H, -CH_2C$H_2$$CH_3$), δ 1.26 (s, 3H, methyl -CH_3), δ 0.88 (t, J = 7.4 Hz, 3H, -CH_3).

MTC-O*i*Bu (M4): (2.5 g, 73% yield). ^{1}H-NMR (400 MHz, $CDCl_3$, 22 °C: δ = 4.57-4.36 (m, 4H, -C$H_a$$H_b$ and -C$H_a$$H_b$), δ 3.94 (d, J = 6.4 Hz, 2H, -OCH_2), δ 1.98-1.88 (m, 1H, -CH(CH_3)$_2$), δ 1.28 (s, 3H, methyl -CH_3), δ 0.90-0.88 (m, 6H, -CH(CH_3)$_2$).

MTC-OHex (M5): (2.2 g, 65% yield)^{1}H-NMR (400 MHz, $CDCl_3$, 22 °C: δ = 4.69-4.16 (m, 6H, -C$H_a$$H_b$, -C$H_a$$H_b$ and -OCH_2), δ 1.68-1.61 (quintet, 2H, -CH_2), δ 1.31-1.29 (m, 9H, methyl -CH_3 and -CH_2), δ 0.88 (t, J = 6.9 Hz, 3H, terminal alkyl CH_3).

Synthesis of Boc-protected random polycarbonate copolymers (P1'-P10')

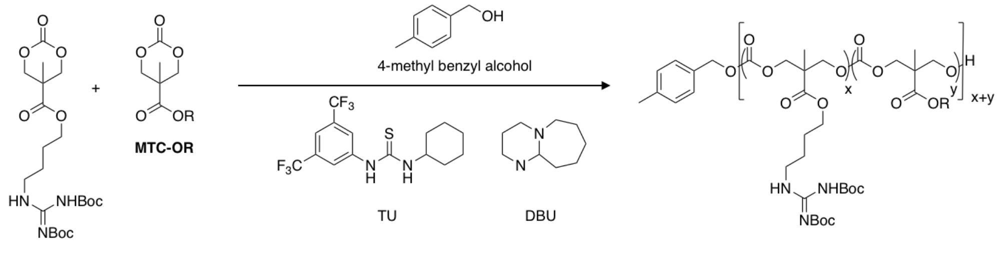

MTC-OBu-BocGua

P(MTC-OBu-BocGua$_x$-MTC-OR$_y$)$_{x+y}$

H1': P(MTC-OBu-BocGua$_{20}$)
P1': P(MTC-OBu-BocGua$_{12}$-MTC-OBn$_6$)$_{18}$
P2': P(MTC-OBu-BocGua$_{14}$-MTC-OEt$_4$)$_{18}$
P3': P(MTC-OBu-BocGua$_{15}$-MTC-OBu$_5$)$_{20}$
P4': P(MTC-OBu-BocGua$_{13}$-MTC-O*i*Bu$_4$)$_{17}$
P5': P(MTC-OBu-BocGua$_{17}$-MTC-OHex$_6$)$_{23}$
P6': P(MTC-OBu-BocGua$_{19}$-MTC-OBn$_3$)$_{22}$
P7': P(MTC-OBu-BocGua$_{16}$-MTC-OEt$_2$)$_{18}$
P8': P(MTC-OBu-BocGua$_{17}$-MTC-OBu$_2$)$_{19}$
P9': P(MTC-OBu-BocGua$_{16}$-MTC-O*i*Bu$_2$)$_{18}$
P10': P(MTC-OBu-BocGua$_{16}$-MTC-OHex$_2$)$_{18}$

The synthesis of **P1'** is given as a representative example. Following the general procedure [1], a mixture of 4-methylbenzyl alcohol (4.88 mg, 0.04 mmol), MTC-OBu-Gua (355.14 mg, 0.75mmol), MTC-OBn (62.56 mg, 0.25 mmol), thiourea (3.70 mg, 0.010 mmol) and DBU (1.52 µL, 0.010 mmol) co-catalyst were reacted for 15 min. The reaction mixture was quenched with 150 mg benzoic acid and purified according to the general procedure to yield **P1'** as a

white solid. ^{1}H NMR (400 MHz, $CDCl_3$, 22 °C): δ 11.50 (s, 11H, N*H*), 8.33 (bs, 11H, N*H*), 7.31-7.26 (m, 36H, Ph -C*H* overlapped with residual $CDCl_3$ peak), 4.27 (m, 61H, $-CH_aH_b$ and $-CH_aH_b$), 4.13 (bs, 23H, $-OCH_2$), 3.43 (bs, 24H, $-CH_2N$), 2.33 (s, 3H, initiator $-CH_3$), 1.69-1.64 (m, 59H, $-CH_2-$), 1.52-1.36 (m, 275H, Boc $-CH_3$), 1.18-1.06 (m, 51H, $-CH_3$). ^{1}H NMR (400 MHz, DMSO, 22°C) δ 7.32-7.15 (m, 30H, Ph -C*H*), 4.20 (m, 56H, $-CH_aH_b$ and $-CH_aH_b$), 4.04 (bs, 20H, $-OCH_2$), 2.28 (s, 3H, initiator $-CH_3$), 1.47-1.36 (m, 203H, Boc $-CH_3$) 1.52 (bs, 33H, BHT), 1.18-1.06 (m, 51H, methyl $-CH_3$).

P2'. ^{1}H NMR (400 MHz, $CHCl_3$, 22 °C): δ 11.49 (s, 11H, N*H*), 8.34-8.31 (m, 13H, N*H*), 4.28 (m, 65H, $-CH_aH_b$ and $-CH_aH_b$), 4.19-4.13 (m, 34H, $-OCH_2$), 3.46-3.42 (m, 25H, -CH_2N), 2.34 (s, 3H, initiator $-CH_3$), 1.72-1.63 (m, 72H, $-CH_2CH_2$), 1.49 (s, 247H, Boc $-CH_3$ and BHT) 1.25-1.20 (m, 70H, methyl $-CH_3$ and alkyl terminal $-CH_3$).

P3'. ^{1}H NMR (400 MHz, $CHCl_3$, 22 °C): δ 11.49 (s, 13H, N*H*), 8.34-8.31 (m, 14H, N*H*), 4.28 (m, 70H, $-CH_aH_b$ and $-CH_aH_b$), 4.16-4.10 (m, 38H, $-OCH_2$), 3.45 (m, 30H, $-CH_2N$), 2.34 (s, 3H, initiator $-CH_3$), 1.69-1.58 (m, 88H, $-CH_2CH_2-$ overlapped with residual H_2O peaks), 1.49-1.33 (m, 286H, Boc $-CH_3$ and BHT), 1.25-1.20 (m, 58H, $-CH_2CH_2-$), 0.92 (t, J = 7.38 Hz, 17H, alkyl terminal $-CH_3$).

P4'. ^{1}H NMR (400 MHz, $CHCl_3$, 22 °C): δ 11.49 (s, 11H, N*H*), 8.34-8.32 (m, 12H, N*H*), 4.28 (m, 56H, $-CH_aH_b$ and $-CH_aH_b$), 4.15-4.12 (m, 24H, $-OCH_2$), 3.46-3.41 (m, 25H, -CH_2N), 2.33 (s, 3H, initiator $-CH_3$), 1.67-1.62 (m, 77H, $-CH_2CH_2-$ and BHT), 1.54-1.43 (m, 227H, Boc $-CH_3$), 1.27-1.19 (m, 52H, methyl $-CH_3$), 0.93-0.88 (m, 25H, $CH(CH_3)_2$).

P5'. ^{1}H NMR (400 MHz, $CHCl_3$, 22 °C): δ 11.50 (s, 12H, N*H*), 8.38 (s, 15H, N*H*), 4.29 (m, 80H, $-CH_aH_b$ and $-CH_aH_b$ and $-OCH_2$ of hydrophobic moiety), 4.16-4.09 (m, 43H, -OCH_2), 3.47-3.42 (m, 33H, $-CH_2N$), 2.34 (s, 3H, initiator $-CH_3$), 1.69-1.59 (m, 98H, $-CH_2CH_2-$ and $-OCH_2CH_2$ of hydrophobic moiety), 1.49 (s, 327H, Boc $-CH_3$), 1.34-1.19 (m, 95H, -$CH_2CH_2CH_2-$ of hydrophobic moiety), 0.88 (t, J = 6.64 Hz, 17H, terminal alkyl $-CH_3$).

P6'. ^{1}H NMR (400 MHz, $CHCl_3$, 22 °C): δ 11.49 (s, 17H, N*H*), 8.33-8.31 (m, 17H, N*H*), 7.34-7.26 (m, 27H, Ph -C*H* overlapped with residual $CDCl_3$ peak), 5.60-5.07 (m, 22H, -OCH_2Ph and BHT), 4.28 (s, 75H, $-CH_aCH_b$ and $-CH_aCH_b$), 4.14-4.13 (m, 34H, $-OCH_2$), 3.44-3.43 (m, 37H, $-CH_2N$), 2.34 (s, 3H, initiator $-CH_3$), 1.49 (s, 340H, Boc $-CH_3$ and BHT), 1.69-1.64 (m, 93H, $-CH_2$), 1.27-1.19 (m, 66H, methyl $-CH_3$). ^{1}H NMR (400 MHz, DMSO, 22°C): δ 7.30-7.15 (m, Ph -C*H*), 4.24-4.06 (m, 112H, $-CH_aCH_b$ and CH_aCH_b and - OCH_2), 2.28 (s, 3H, initiator $-CH_3$), 1.54 (BS, 59H, $-CH_2$), 1.45-1.36 (m, 314H, Boc $-CH_3$ and BHT), 1.17-1.07 (m, 60H, methyl $-CH_3$).

P7'. ^{1}H NMR (400 MHz, $CHCl_3$, 22 °C): δ 11.49 (s, 15H, N*H*), 8.33-8.31 (m, 15H, N*H*), 4.33-4.28 (m, 63H, -CH_aCH_b and -CH_aCH_b), 4.16-4.13 (m, 34H, -OCH_2), 3.46-3.42 (m, 32H, -CH_2N), 2.34 (s, 3H, initiator -CH_3), 1.69-1.61 (m, 80H, -CH_2CH_2), 1.49 (s, 296H, Boc -CH_3 and BHT), 1.25-1.24 (m, 54H, methyl -CH_3).

P8'. ^{1}H NMR (400 MHz, $CHCl_3$, 22 °C): δ 11.49 (s, 17H, N*H*), 8.34-8.32 (m, 17H, N*H*), 4.33-4.23 (m, 69H, -CH_aCH_b and -CH_aCH_b), 4.16-4.09 (m, 43H, -OCH_2), 3.47-3.42 (m, 34H, -CH_2N), 2.33 (s, 3H, initiator -CH_3), 1.72 -1.63 (m, 95H, -CH_2), 1.49 (s, 315H, Boc -CH_3 and BHT), 1.27-1.19 (m, 66H, methyl -CH_3 overlapped with residual diethyl ether peak), 0.91 (t, J = 7.34 Hz, 7H, terminal alkyl -CH_3).

P9'. ^{1}H NMR (400 MHz, $CHCl_3$, 22 °C): δ 11.49 (s, 16H, N*H*), 8.34-8.31 (m, 16H, N*H*), 4.28 (bs, 66H, -CH_aCH_b and -CH_aCH_b), 4.16-4.13 (m, 30H, -OCH_2), 3.46-3.42 (m, 32H, -CH_2N), 2.34 (s, 3H, initiator -CH_3), 1.72 -1.61 (m, 69H, -CH_2), 1.49 (s, 300H, Boc -CH_3 and BHT), 1.29-1.19 (m, 56H, methyl -CH_3), 094-0.88 (m, 13H, -$CH_2(CH_3)_2$).

P10'. ^{1}H NMR (400 MHz, $CHCl_3$, 22 °C): δ 11.49 (s, 15H, N*H*), 8.34-8.31 (m, 15H, N*H*), 4.28 (bs, 62H, -CH_aCH_b and -CH_aCH_b), 4.16-4.13 (m, 34H, -OCH_2), 3.46-3.42 (m, 31H, -CH_2N), 2.34 (s, 3H, initiator -CH_3), 1.72 -1.64 (m, 87H, -CH_2), 1.49 (s, 282H, Boc -CH_3), 1.35-1.20 (m, 65H, methyl -CH_3 and alkyl -CH_2 of hydrophobic moiety), 0.88 (t, J = 6.6 Hz, 5H, -CH_3).

Synthesis of deprotected random polycarbonate copolymers (P1-P10)

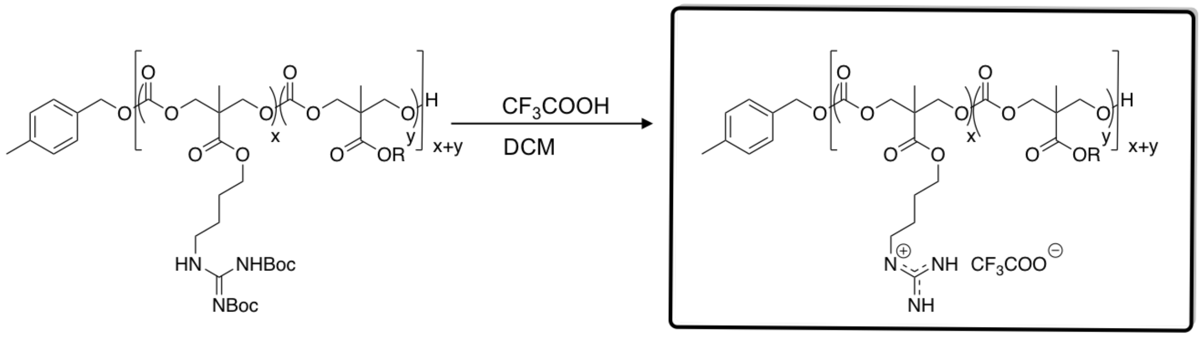

P(MTC-OBu-BocGua$_x$-MTC-OR$_y$)$_{x+y}$

P(MTC-OBu-Gua$_x$-MTC-OR$_y$)$_{x+y}$

H1: P(MTC-OBu-Gua$_{20}$)
P1: P(MTC-OBu-Gua$_{12}$-MTC-OBn$_6$)$_{18}$
P2: P(MTC-OBu-Gua$_{14}$-MTC-OEt$_4$)$_{18}$
P3: P(MTC-OBu-Gua$_{15}$-MTC-OBu$_5$)$_{20}$
P4: P(MTC-OBu-Gua$_{13}$-MTC-O*i*Bu$_4$)$_{17}$
P5: P(MTC-OBu-Gua$_{17}$-MTC-OHex$_5$)$_{22}$
P6: P(MTC-OBu-Gua$_{19}$-MTC-OBn$_3$)$_{22}$
P7: P(MTC-OBu-Gua$_{16}$-MTC-OEt$_2$)$_{18}$
P8: P(MTC-OBu-Gua$_{17}$-MTC-OBu$_2$)$_{19}$
P9: P(MTC-OBu-Gua$_{16}$-MTC-O*i*Bu$_2$)$_{18}$
P10: P(MTC-OBu-Gua$_{16}$-MTC-OHex$_2$)$_{18}$

The synthesis of **P1** is given as a representative example. Briefly [1], all the polymers (150 mg) was dissolved in DCM (9 mL) and trifluoroacetic acid (1 mL), and stirred at room temperature overnight. The removal of solvents in vacuo yielded a yellow sticky solid as the deprotected guanidinium-functionalised polymer in quantitative yields. Subsequently, the polymer was solubilised in water and lyophilised to yield a white solid **P1.** ^{1}H NMR (400 MHz, CD_3OD, 22 °C): δ 7.35 (m, 20H, Ph -C*H*), 5.16 (m, 8H, -C*H*$_2$), 4.30-4.28 (m, 61H, -C*H*$_a$CH$_b$ and -CH$_a$C*H*$_b$ overlapped with residual H_2O peak), 4.17-4.16 (m, 27H, -OC*H*$_2$), 3.22-3.20 (m, 26H, -C*H*$_2$N), 1.71-1.66 (m, 55H, -C*H*$_2$), 1.28-1.14 (m, 57H, methyl -C*H*$_3$).

P2. ^{1}H NMR (400 MHz, CD_3OD, 22 °C): δ 4.32 (s, 77H, -C*H*$_a$CH$_b$ and -CH$_a$C*H*$_b$ overlapped with residual H_2O peak), 4.23-4.18 (m, 41H, -OC*H*$_2$), 3.26-3.23 (m, 28H, -C*H*$_2$N), 1.77-1.67 (m, 63H, -C*H*$_2$), 1.28-1.25 (m, 69H, -C*H*$_3$), 1.21-1.17 (m, 11H, terminal alkyl -C*H*$_3$).

P3. ^{1}H NMR (400 MHz, CD_3OD, 22 °C): δ 4.31 (s, 62H, -C*H*$_a$CH$_b$ and -CH$_a$C*H*$_b$ overlapped with residual H_2O peak), 4.19-4.17 (m, 30H, -OC*H*$_2$), 3.25-3.22 (m, 30H, -C*H*$_2$N), 1.74-1.68 (m, 63H, -C*H*$_2$), 1.29-1.26 (m, 44H, -C*H*$_3$), 1.21-1.17 (m, 9H, -C*H*$_3$).

P4. ^{1}H NMR (400 MHz, CD_3OD, 22 °C): δ 4.35-4.30 (m, 63H, -C*H*$_a$CH$_b$ and -CH$_a$C*H*$_b$ overlapped with residual H_2O peak), 4.20-4.12 (m, 31H, -OC*H*$_2$), 3.24-3.20 (m, 26H, -C*H*$_2$N),

1.75-1.65 (m, 57H, -CH_2), 1.28-1.15 (m, 66H, -CH_3), 0.94 (d, J = 6.7 Hz 25H, terminal alkyl -CH_3).

P5. ^{1}H NMR (400 MHz, CD_3OD, 22 °C): δ 4.32 (m, 88H, C$H_a$$H_b$ and C$H_a$$H_b$ overlapped with residual H_2O peak), 4.18 (m, 47 H, -OCH_2-), 3.24 (m, 34H, -CH_2N-), 1.77-1.64 (m, 84H, -CH_2-), 1.41-1.35 (m, 31H, -CH_2 of hexyl side chain), 1.22 (bs, 65H, -CH_3-), 0.93 (t, J = 6.64 Hz, 17H, terminal -CH_3 of hexyl side chain).

P6. ^{1}H NMR (400 MHz, CD_3OD, 22 °C): δ4.32 (s, 95H, -CH_aCH_b and -CH_aCH_b overlapped with residual H_2O peak), 4.12-4.05 (m, 52H, -OCH_2), 3.17-3.13 (m, 38H, -CH_2N), 1.67-1.52 (m, 95H, -CH_2 overlapped with BHT), 1.19-1.11 (m, 81H, methyl -CH_3 overlapped with residual diethyl ether peak).

P7. ^{1}H NMR (400 MHz, CD_3OD, 22 °C): δ 4.32 (s, 68H, -CH_aCH_b and -CH_aCH_b), 4.21-4.16 (m, 36H, -OCH_2), 3.26-3.22 (m, 32H, -CH_2N), 1.76-1.61 (m, 71H, -CH_2), 1.28-1.20 (m, 63H, -CH_3), 1.17-1.16 (m, 6H, terminal alkyl -CH_3).

P8. ^{1}H NMR (400 MHz, CD_3OD, 22 °C): δ 4.35-4.26 (m, 73H, -CH_aCH_b and -CH_aCH_b), 4.19-4.14 (m, 44H, -OCH_2), 3.24-3.29 (m, 34H, -CH_2N), 1.75-1.61 (m, 81H, -CH_2), 1.26-1.15 (m, 69H, methyl -CH_3 and -CH_2 of hydrophobic moiety), 0.97-0.93 (m, J = 7.26 Hz, 7H, terminal alkyl -CH_3).

P9. ^{1}H NMR (400 MHz, CD_3OD, 22 °C): δ 4.30 (m, 65H, -CH_aCH_b and -CH_aCH_b), 4.19-4.16 (m, 32H, -OCH_2), 3.24-3.21 (m, 32H, -CH_2N), 1.75-1.65 (m, 69H, -CH_2), 1.26-1.16 (m, 53H, methyl -CH_3), 0.95-0.93 (m, 12H, -CH_2(CH_3)$_2$).

P10. ^{1}H NMR (400 MHz, CD_3OD, 22 °C): δ 4.31 (m, 65H, -CH_aCH_b and -CH_aCH_b), 4.21-4.13 (m, 36H, -OCH_2), 3.26-3.22 (m, 32H, -CH_2N), 1.18-1.64 (m, 70H, -CH_2), 1.44-1.17 (m, 73H, methyl -CH_3 and -CH_2CH_2CH_2 of hydrophobic moiety), 0.98-0.93 (m, 10H, terminal alkyl -CH_3).

Synthesis of AF488-conjugated polymers (AF488-H1 and AF488-P5)

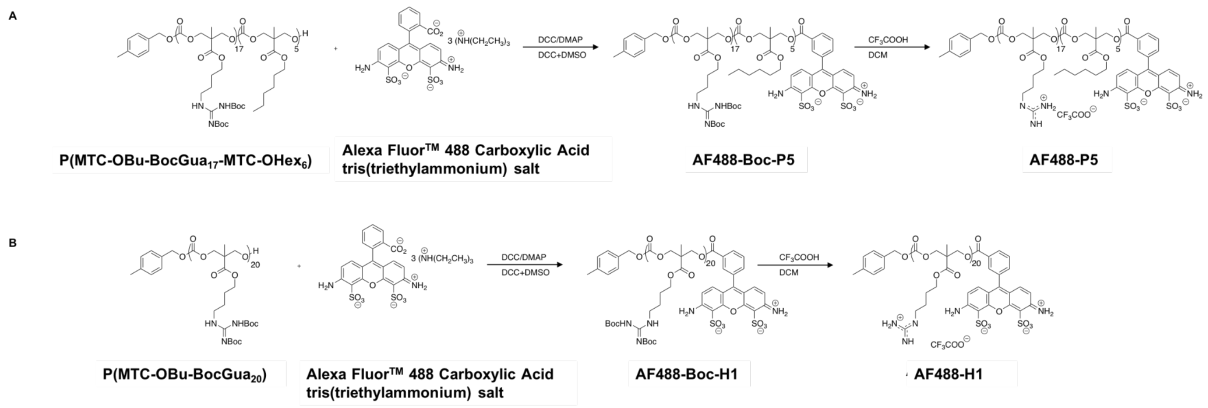

Scheme S2. Synthetic procedure and chemical structure of (A) **AF488-P5** and (B) **AF488-H1** conjugates.

The synthesis of the fluorescent dye-labeled polymer was achieved by grafting Alexa Fluor 488 dye to the chain end of the polymer, and then the protecting group of pending ethyl guanidine group on the polymer was removed by addition of trifluoroacetic acid (TFA). The synthesis of **AF488-P5** is given as a representative example. Briefly, **P5** (34.9 mg, Mn ~ 7,660 Da, 0.00455 mmol) and Alexa Fluor 488 dye (5 mg, 0.0091 mmol) were allowed to react in a solution containing 6 mL of dry DMSO and 1 mL of dry DCM in the presence of a catalytic amount of 4-(dimethylamino)pyridine (DMAP, 0.9 mg). Once the solution turned homogeneous, 36.4 μL of dicyclohexylcarbodiimide (DCC) solution (1.0 M in DCM, 0.036 mmol) was added and allowed to stir for 48 hrs. The resulting mixture was dialyzed four times against DMSO (MWCO 2 kDa) and 5 times against the mixture of MeOH and DCM (volume ratio 1:1) sequentially. Subsequently, the solvent was removed completely and the resulting solute was re-dissolved in 12 mL of dry DCM and 2 mL of TFA, and left to stir overnight. After that, the solvents were rotavap dried, and the residue obtained was dissolved in MeOH and dialyzed against a mixture containing equal volume of MeOH and DCM thrice (MWCO 1 kDa). The solvent in the dialysis bag was removed completely and the resulting solute was freeze-dried, yielding **AF488-P5** as a brightly orange powder.

Supplementary Data

Cell viability curves for MCF-7 and SW480 after 24 hour incubation with the polymers

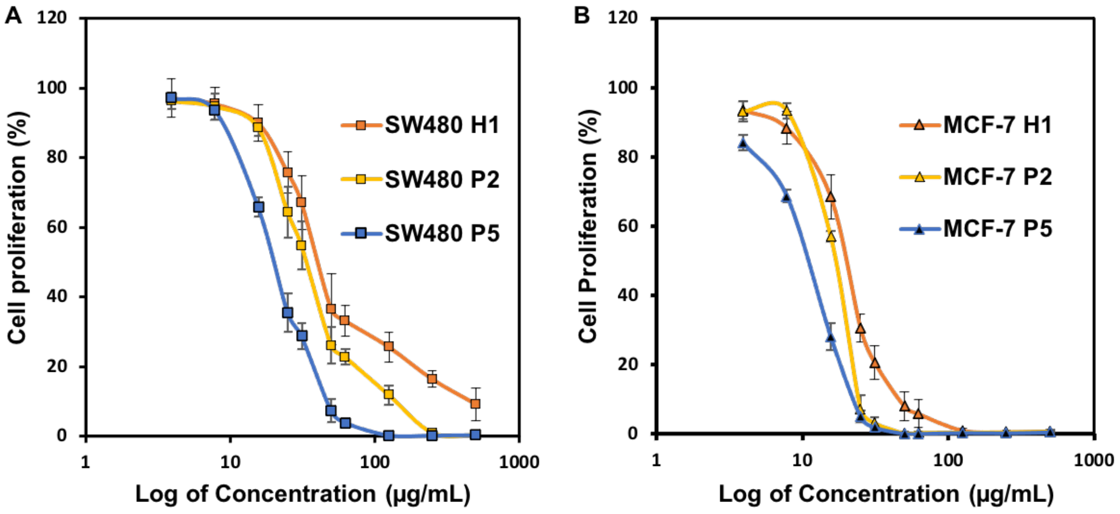

Figure S1. *In vitro* anticancer activity of polymers against (A) MCF-7 breast cancer cell line and (B) SW480 colorectal cancer cell lines after 24 h incubation. Results were expressed as the mean ± standard deviation shown by the error bars (n = 3).

Cell viability curves for HK2 after 24 hour incubation with the polymers

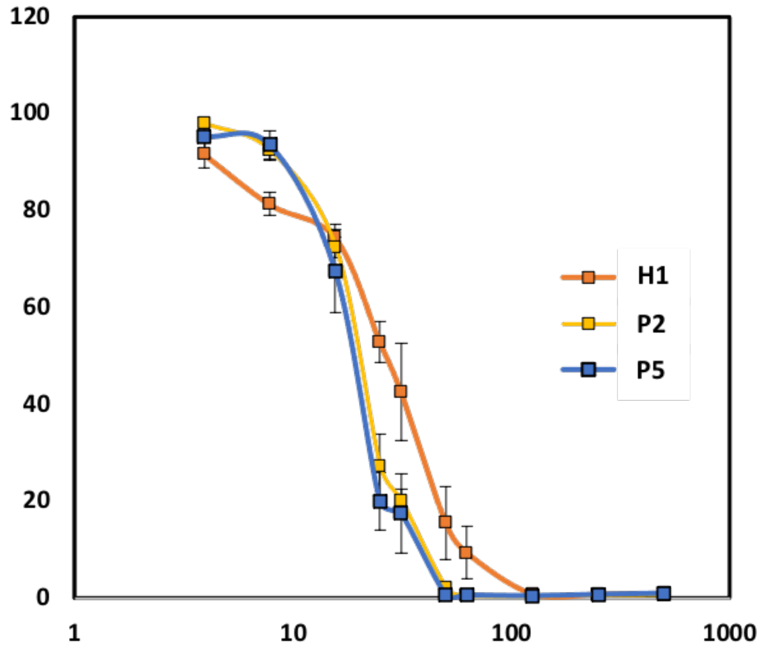

Entry	Polymer	IC_{50} ± SD (ppm)
1	**H1**	26±3.2
2	**P2**	20±0.0
3	**P5**	19±1.0

Figure S2. Cytotoxicity of polymers against HK2 healthy kidney cell line after 24 h incubation. Results were expressed as the mean ± standard deviation shown by the error bars (n = 3).

Characterization of Boc-protected polymers using SEC

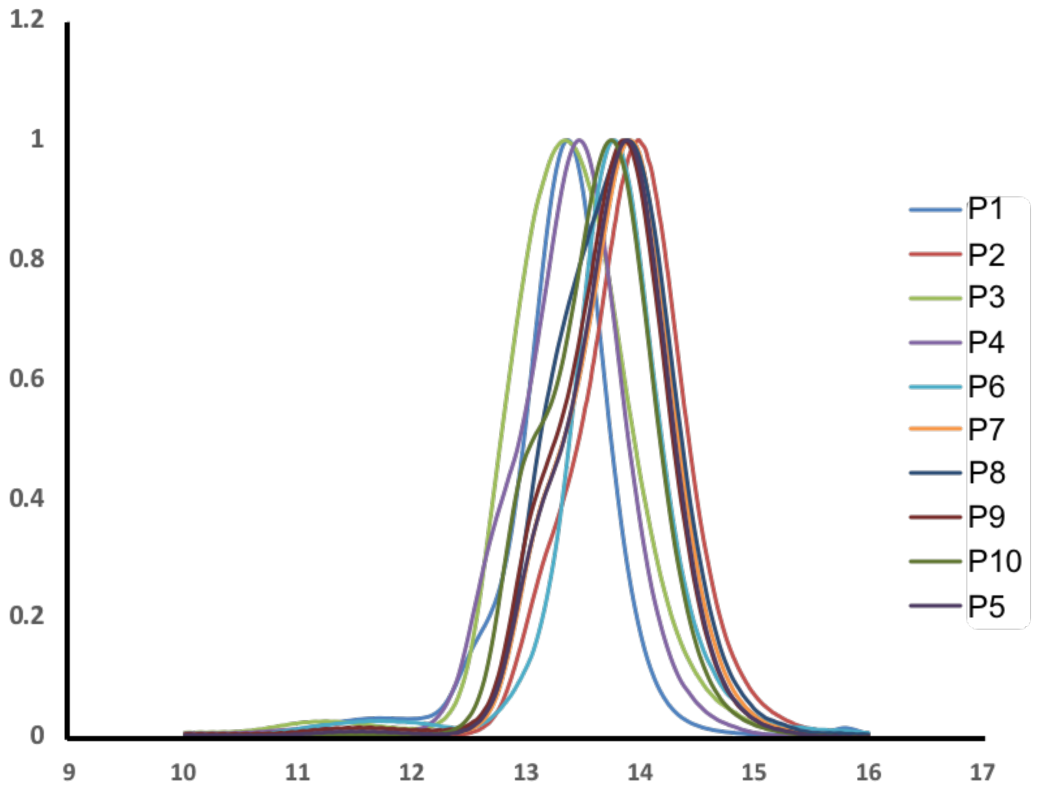

Figure S3. SEC chromatograms of representative polycarbonates prior to deprotection.

References

1. Chin W, Zhong G, Pu Q, Yang C, Lou W, De Sessions P F, et al. A macromolecular approach to eradicate multidrug resistant bacterial infections while mitigating drug resistance onset. Nat Commun. 2018; 9: 917.
2. Tan E W P, Hedrick J L, Arrechea P L, Erdmann T, Kiyek V, Lottier S, et al. Overcoming barriers in polycarbonate synthesis: A streamlined approach for the synthesis of cyclic carbonate monomers. Macromolecules. 2021; 54: 1767-74.
3. Pratt R C, Nederberg F, Waymouth R M, Hedrick J L. Tagging alcohols with cyclic carbonate: A versatile equivalent of (meth)acrylate for ring-opening polymerization. Chem Commun. 2008: 114-16.
4. Thongsomboon W, Sherwood M, Arellano N, Nelson A. Thermally induced nanoimprinting of biodegradable polycarbonates using dynamic covalent cross-links. ACS Macro Lett. 2013; 2: 19-22.
5. Tempelaar S, Mespouille L, Coulembier O, Dubois P, Dove A P. Synthesis and post-polymerisation modifications of aliphatic poly(carbonate)s prepared by ring-opening polymerisation. Chem Soc Rev. 2013; 42: 1312-36.
